# Universal topology of exceptional points in nonlinear non-Hermitian systems

**DOI:** 10.1038/s41467-026-72854-2

**Published:** 2026-05-20

**Authors:** Nai-Hang Kwong, Jan Wingenbach, Laura Ares, Jan Sperling, Xuekai Ma, Stefan Schumacher, Rolf Binder

**Affiliations:** 1 Wyant College of Optical Sciences, University of Arizona, Tucson, AZ USA; 2 Department of Physics and Center for Optoelectronics and Photonics Paderborn (CeOPP), Paderborn University, Paderborn, Germany; 3 Institute for Photonic Quantum Systems (PhoQS), Paderborn University, Paderborn, Germany; 4 Department of Physics, University of Arizona, Tucson, AZ USA

**Keywords:** Optical physics, Photonic devices, Nonlinear optics

## Abstract

Exceptional points are non-Hermitian degeneracies where eigenvalues and eigenvectors coalesce, giving rise to unusual physical effects across scientific disciplines. The concept of exceptional points has recently been extended to nonlinear physical systems. We theoretically demonstrate a universal topology in the nonlinear parameter space for a large class of physical systems that support second-order exceptional points in the linear regime. Knowledge of this topology (called elliptic umbilic singularity in bifurcation theory) deepens our understanding of second-order linear exceptional points, which here emerge as coalescence of four nonlinear eigenvectors. This helps guide future experimental discovery of nonlinear exceptional points and their classification, establish rigorous bounds of sensitivity enhancement of exceptional points in nonlinear systems, and helps envision and optimize technological applications of nonlinear exceptional points. Our theoretical approach is general and can be extended to nonlinear perturbations of third-order and higher-order exceptional points.

## Abstract (web summary)

Exceptional points (EPs) are non-Hermitian degeneracies across various physical systems, including nonlinear ones. Here, by using catastrophe theory, the authors demonstrate a universal topology applicable to nonlinear systems that support second-order linear EPs. This framework provides a useful guide for future experimental applications of nonlinear EPs and their classification.

## Introduction

Exceptional points (EPs), which are spectral degeneracies of non-Hermitian systems at which both eigenvalues and eigenvectors coalesce (mathematical definitions and generalizations are in refs. ^1–3^), have been increasingly explored across a broad range of scientific areas, including anharmonic oscillators^4^, diffraction optics^5^, quantum chaos^6,7^, conventional lasers^8,9^, waveguides and photonic systems^10–13^, atomic gases, condensates and high-Q cavity atomic lasers^14–16^, polariton lasers, microlasers and polariton condensates^17–25^, non-Hermitian Bose–Hubbard models^26^, critical fluctuations and fluctuation spectra^27,28^, and sensing applications^29–34^, non-Hermitian parity-time (PT) symmetric quantum systems^35–44^, and quantum state control and squeezing^45,46^. EPs have also been studied in the context of topological insulators and other topological systems (for example refs. ^47–56^).

While in most of these physical realizations EPs emerge as eigenvalues *λ* of a linear non-Hermitian matrix *M*, in other words from the eigenvalue problem *M***x** = *λ***x**, more recent studies of EPs include nonlinear physical systems, such as coupled optical or electric resonators^57^, models of population dynamics^58^, as well as polariton condensates in microresonators. In these systems, the complex interplay of nonlinear and non-Hermitian physics can lead to counterintuitive phenomena, such as shifts^59–62^ and rotations^62^ of the EP in parameter space through blueshifts and saturable gain. Moreover, mode switching can be realized in bistable regions near the EP^63^. In refs. ^62,64^, it was shown that nonlinearity can alter the eigenvalue splitting in the vicinity of an EP and highly sensitive EP-based sensors have been realized in nonlinear non-Hermitian systems^65–67^. The underlying eigenvalue problem here may be nonlinear in the sense that the matrix *M* may depend on the eigenvalue *λ* (for example ref. ^68^), the eigenvector **x** (for example ref. ^69^), or both. In some instances, the nonlinear dependence on the eigenvector can be interchanged by that on the eigenvalue, for example ref. ^34^. Other kinds of nonlinear eigenvalue problems are discussed in ref. ^70^. In the following, we concentrate on the case where the nonlinear eigenvalue problem is formulated as a matrix equation where the matrix depends on the eigenvector. For example, the energies in an optical microcavity, which are described by the diagonal elements of *M*, can shift with the intensity of the light field described by **x**, hence *M*(**x**)**x** = *λ***x**. In the following, we refer to EPs that are solutions of a linear eigenvalue problem as ‘linear EPs,’ and those solving a nonlinear eigenvalue problem are called ‘nonlinear EPs.’

The wide range of physical systems in which EPs can occur raises the question whether there exist any guiding principles that help clarify the occurrence of EPs as the physical parameters (in particular the nonlinearity) entering the matrix *M* are varied.

In this paper, we demonstrate that important insight can be obtained on the distribution of EPs in parameter space, especially for nonlinear eigenvalue problems, with implications for photonic systems with Kerr-type nonlinearity, atomic^71,72^ and polariton condensates^73^, superfluids^74^ and coupled ring or micropillar optical^75^ and electric resonators^66^. Additional novel applications, such as robust wireless power transfer and enhanced frequency-comb generation are discussed in ref. ^76^. We show that a powerful way to do this is to consider the behavior of eigenvectors around EPs as a bifurcation problem and make use of established mathematical results in catastrophe theory.

**Fig. 1:**
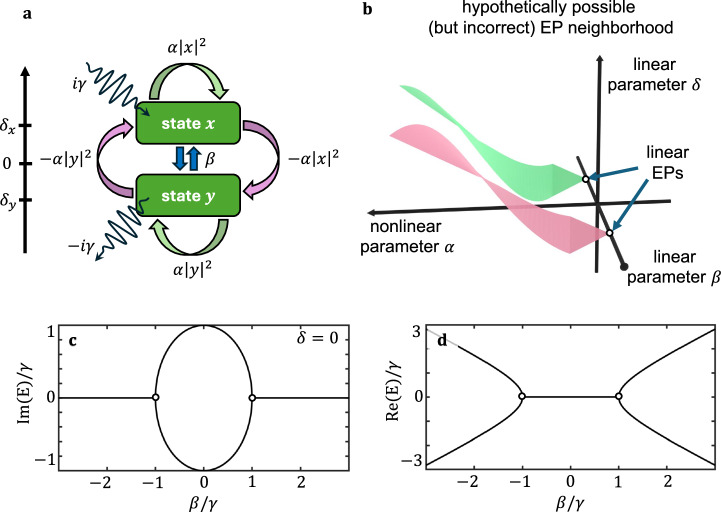
Non-Hermitian two-state system. **a** Sketch of a non-Hermitian and nonlinear two-state (or two-mode) system, with linear coupling *β*, energy splitting 2*δ* = *δ*_*x*_ − *δ*_*y*_, and loss/gain difference 2*γ*, cf. Eq. (2). **b** Sketch of hypothetical location of exceptional points (EPs) as a function of physical parameters. Without a nonlinearity, *α* = 0, isolated linear EPs are at (*β*, *δ*) = ( ± *γ*, 0); in the nonlinear regime, *α* ≠ 0, EPs are implied on the colored surfaces. To illustrate the problem our study is solving, the surfaces we show here are one of infinitely many hypothetically possible (but generally wrong) EP neighborhoods. The correct geometric shape is shown in Fig. 2a. **c** and **d** show the eigenvalues in the linear case (*α* = 0) with two isolated EPs at (*β*, *δ*) = ( ± *γ*, 0).

We illustrate this rigorous procedure with an analysis of nonlinear parameter-dependent 2 × 2 matrices, schematically shown in Fig. 1, and show that a broad class of physically different nonlinear systems share the same EP neighborhood geometry that fixes one (and only one) topology, namely a cone-like structure (with deltoid cross section) that consists of three fold-bifurcation surfaces attached to each other at cusp-bifurcation ribs. As a corollary, we find the possible numbers of solutions (eigenvalues) in each region of parameter space. All these insights are based on one single equation (namely that for the so-called Lyapunov potential). Our findings can be used in future studies to establish rigorous bounds on the sensitivity enhancement of EPs in nonlinear systems. It also provides guardrails against possibly wrong conclusions, illustrated in Fig. 1b, that might be obtained from limited experimental observations or approximate theories.

To solve the highly sophisticated problem outlined above, we utilize the general theorems of a branch of bifurcation theory called catastrophe theory^77–82^. Roughly speaking, the framework of catastrophe theory describes the shapes of singularity sets – or phase boundaries in nonequilibrium phase diagrams, to use a physicist’s language – related to singularities of potential functions *V*_*L*_ (Lyapunov potentials). The word catastrophe is used for the sudden qualitative change or bifurcation at a ‘phase boundary.’ Moreover, it lists all possible shapes (‘elementary catastrophes’) for cases in which the number of variables (often called state variables) and the number of control parameters (external parameters) are relatively small, see Supplementary Table 1 in the Supplementary Information.

Examples of systems that have been analyzed by catastrophe theory include dynamical systems obeying equations of motion of the form ${\dot{{\bf{x}}}}=-{\rm grad}\{{V}_{L}({{\bf{x}}})\}$, where the phase boundaries in the control parameter space follow from the assumption of degenerate stationary states (degenerate critical points), corresponding to ${\rm grad}\{{V}_{L}({{\bf{x}}})\}=0$ and $\det \{h\}=0$, where *h* denotes the Hessian matrix of *V*_*L*_. Similarly, in optics, the solution of the Helmholtz equation, which describes the stationary solutions of a second-order time-differential equation, in contrast to the example of a first-order time-differential equation mentioned above, can be evaluated with the method of stationary phase and stationary points, as well as caustics occurring when two or more stationary points coalesce (p. 336 of ref. ^83^). This identifies rainbows and other optical phenomena as diffraction catastrophe, specifically fold and cusp bifurcations; see refs. ^83–85^ and Ch. 12 of ref. ^82^ for details. Higher bifurcations (catastrophes), such as the so-called elliptic umbilic, have also been studied in the context of optical diffraction^85,86^. Many other areas of application of catastrophe theory can be found in refs. ^7,11,77,80–82,87–90^. This further extends to studies in biology, social sciences, and economics, with various degrees of acceptance because low-dimensional dynamical-systems models are not always appropriate for the simulation of complex systems in those sciences^88,90^. In contrast, in many areas of physics low-dimensional models and eigenvalue problems (for example two–level models) are well established.

In this study, we aim to understand the topological structure of the EP set around the linear EP in parameter space when nonlinearity in the matrix is turned on. Our strategy of utilizing catastrophe theory is as follows (more details are given in Methods and Supplementary Note 2 of the Supplementary Information). The layout of the catastrophe theory formulation consists of a state variable space and a control parameter space. The eigenvector elements of the nonlinear matrix are considered as values of state variables determined by the eigenvector equations, and the coordinates of the control parameter space are the parameters in the matrix. A Lyapunov potential function is constructed under the condition that its critical points (in state variable space) are equal to the eigenvector elements. The coalescing eigenvectors are accordingly given by the degenerate critical points of the Lyapunov potential. The set of degenerate critical points forms a hyper-surface in the combined state variable space and parameter space. The projection of this hyper-surface onto the parameter space yields the set of EPs in parameter space.

To obtain a topological characterization of the nonlinear EP set in parameter space around the linear EP, we Taylor expand the Lyapunov potential in both the state variables and the parameters about this point. This allows us to match our Lyapunov potential locally with one of the known catastrophes. See, for example, p. 137 of ref. ^80^ and p. 11 of ref. ^81^ for a list of the ‘elementary catastrophes’, which is also reproduced as Supplementary Table 1 in the Supplementary Information. We find that, for the broad class of nonlinear 2 × 2 matrices considered here, the matched catastrophe is the elliptic umbilic. Consequently, the EP sets (in parameter space) of these matrices share a common topological structure which is that of the set of degenerate critical points of this catastrophe.

## Results

In the following, we present our approach for a large class of 2 × 2 matrices and analyze the EP structure in parameter space. We find that, for all matrices in this class, the linear EPs are the organizing points of an elliptic umbilic singularity. We stress that, in addition to the matrices discussed below, there may be other 2 × 2 matrices with elliptic umbilic nonlinear EP structures that we have not yet considered.

In the next subsection, *Example: quadratic nonlinearity*, we begin by illustrating our findings using a specific example, namely a symmetric matrix with Kerr-like nonlinearity, and in the following subsections we discuss extensions and generalizations of that example. All examples discussed below belong to the class of matrices that can be written in the following form (now using the symbol *H* as commonly used in the context of non-Hermitian Hamiltonians)

1 $$H=\left(\begin{array}{cc}\delta+i\gamma+\alpha F(w) & b\\ c & -\delta -i\gamma -\alpha F(w)\end{array}\right)$$

with $\delta,\gamma,\in {\mathbb{R}}$, $b,c,\alpha \in {\mathbb{C}}$, and the nonlinearity *F*(*w*) being a real-valued analytic function of the eigenvector parameter *w* in the vicinity of the linear EP. Specifically, the real-valued variable *w* is related to the (right) eigenvector of *H*, which, normalized to 1, is written as ${[x \, \, y]}^{T}=(1/A){[1\, \, \widetilde{w}]}^{T}$, $\widetilde{w}=w{e}^{i\theta }$, $A=\sqrt{1+{w}^{2}}$. The matrix in Eq. (1) is a useful starting point for our analysis. The fact that *H* is traceless, in other words that the average of the diagonal elements is zero, is not a restriction. Below, in the subsection *Extension beyond quadratic nonlinearities*, we will discuss in more detail the procedure of how to remove the average of the diagonal elements without changing the eigenvectors.

### Example: quadratic nonlinearity

In order to give a concrete example, in this section we illustrate our approach for symmetric 2 × 2 matrices with a second–order linear EP and a nonlinearity that is a quadratic function of the eigenvector variable *w* defined above (Kerr-like nonlinearity). Furthermore, we assume that at the EP, where *w* = 1, the lowest-order Taylor expansion of the nonlinearity function yields *F*(1) = 0 and ${F}^{{\prime} }(1)\ne 0$. We write the nonlinear eigenvalue problem as

2 $$H\left[\begin{array}{c}x\\ y\end{array}\right]=\left[\begin{array}{cc}a & \beta \\ \beta & -a\end{array}\right]\left[\begin{array}{c}x\\ y\end{array}\right]=E\left[\begin{array}{c}x\\ y\end{array}\right]\,,$$

where $a=\delta+i\gamma+\alpha (| x{| }^{2}-| y{| }^{2})$, $\alpha,\beta,\gamma,\delta \in {\mathbb{R}}$ are parameters, and all variables and parameters are assumed scaled and unitless. This parametric form is chosen for ease of analysis and the specific form of the nonlinearity includes a broad range of physical systems^66,71–75^; the restrictions can be relaxed and the method is still applicable (see Sec. 2.2 and Supplementary Note 1 of the Supplementary Information for details). In the linear limit, the two states are separated by 2*δ* in frequency, and one state gains and the other dissipates at equal rates ∣*γ*∣, Fig. 1c,d

Eliminating the eigenvalue *E* from Eq. (2) reduces the matrix equation to a nonlinear equation in the complex eigenvector element $\widetilde{w}$, the real and imaginary parts of which are

3 $$2\alpha \left[\frac{1-{w}^{2}}{1+{w}^{2}}\right]+2\delta+\beta \left[w-\frac{1}{w}\right]\cos \theta=0,$$

4 $$2\gamma+\beta \left[w+\frac{1}{w}\right]\sin \theta=0.$$

Formulating the eigenvalue problem this way enables the map to the function singularity problem to be constructed. In this context, we call (*w*, *θ*) the state variables and (*α*, *β*, *γ*, *δ*) the control parameters. The first step in the map is to construct a Lyapunov function *V*_*L*_(*w*, *θ*, *α*, *β*, *γ*, *δ*) such that its critical point equations in state variable space, ∂*V*_*L*_/∂*w* = 0, ∂*V*_*L*_/∂*θ* = 0, are the same as the eigenvector equations (3) and (4). It can be directly verified that the Lyapunov function

5 $$\begin{array}{rcl}{V}_{L}(w,\theta,\alpha,\beta,\gamma,\delta )=-2\alpha {\mathrm{ln}}\left[g/2\right]+2\delta {\mathrm{ln}}w+\beta g\cos \theta -2\gamma [\theta -\frac{3\pi }{2}]\,,\end{array}$$

with *g* = *w* + *w*^−1^, satisfies this requirement.

To see the connection with catastrophe theory, we need to consider *V*_*L*_ in the vicinity of the linear EPs. Since *V*_*L*_ only has terms that are linear in the control parameters, we can reduce the dimensions of the parameter space by rescaling by one of the parameters, which we choose to be *γ*. The parameter space for the EP analysis, (*α*/*γ*, *β*/*γ*, *δ*/*γ*), is thus three-dimensional. In the linear limit (*α*/*γ* = 0), the matrix *H* has two separate EPs (bifurcation points) in the (*β*/*γ*, *δ*/*γ*) plane at (*β*/*γ*, *δ*/*γ*) = ( ± 1, 0). The coalescing eigenvector element (degenerate critical point) is (*w*, *θ*) = (1, 3*π*/2) for *β*/*γ* = 1 and (*w*, *θ*) = (1, *π*/2) for *β*/*γ* = − 1. Since the critical point behavior around the two linear EPs is related by reflection symmetry about the plane *β* = 0, it suffices to analyze one, *β*/*γ* = 1, of the two. We choose, for convenience, the (*w*, *θ*)-independent terms in Eq. (5) for *V*_*L*_ to shift *V*_*L*_ to zero at the *β*/*γ* = 1 linear EP. We firstly consider the properties of the nonlinear EP set in the immediate neighborhood of this linear EP. We write *r* = *w* − 1, *ϕ* = *θ* − 3*π*/2, ${\beta }^{{\prime} }/\gamma=\beta /\gamma -1$ and denote by ${V}_{EU}(r,\phi,\alpha /\gamma,{\beta }^{{\prime} }/\gamma,\delta /\gamma )$ the lowest-order terms in an expansion in $(r,\phi,\alpha /\gamma,{\beta }^{{\prime} }/\gamma,\delta /\gamma )$ of *V*_*L*_. Then, we find

6 $${V}_{EU}(r,\phi,\alpha /\gamma,{\beta }^{{\prime} }/\gamma,\delta /\gamma )=-\left[\frac{{\phi }^{3}}{3}-\phi {r}^{2}+\frac{\alpha }{\gamma }{r}^{2}-2\frac{{\beta }^{{\prime} }}{\gamma }\phi -2\frac{\delta }{\gamma }r\right].$$

The form of Eq. (6) matches that of a universal unfolding of the *D*_−4_ singularity, also called elliptic umbilic singularity, around the linear EP. Its degenerate critical points and bifurcations, which are well understood, are obtained as simultaneous solutions to three equations: ∂*V*_*EU*_/∂*r* = 0, ∂*V*_*EU*_/∂*θ* = 0, and $\det \{h\}=0$, where *h* is the Hessian matrix of *V*_*EU*_. The corresponding set of three equations, with *V*_*L*_ taking the place of *V*_*EU*_, give the EP set in the whole parameter space (the singularity set). Since there are five unknowns, *w*, *θ*, *α*/*γ*, ${\beta }^{{\prime} }/\gamma$, *δ*/*γ*, the solution set of the three equations forms a 2D surface in the 5D combined state variable and parameter space. The projection of this solution surface onto the parameter space is the singularity set. Algebraic detail of the solution set can be found in Supplementary Note 3 of the Supplementary Information.

**Fig. 2:**
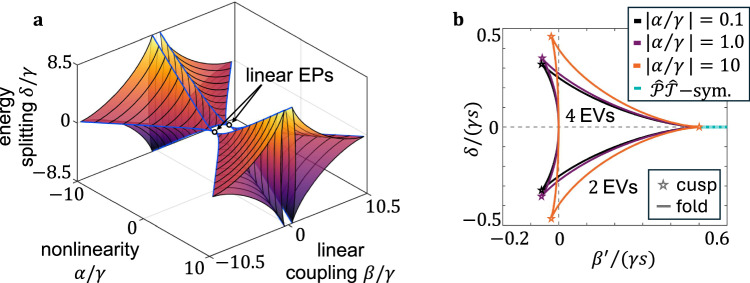
Elliptic umbilic singularity set. **a** Surface of exceptional point (EP) locations, shown as shaded areas, as a function of physical parameters (singularity set) explained in Fig. 1. In the weakly nonlinear regime, *α* ≪ *γ*, the shape approaches that of an elliptic umbilic bifurcation (`catastrophe'). The shape of each singularity set consists of two infinite three-cusped conical surfaces, the apices of which meet at the two linear EPs (*α*, *β*, *δ*) = (0, ± *γ*, 0). At large *α*, the three-cusped shape remains but is deformed guaranteeing that the two cone-like surfaces originating at *β* = ± 1 do not overlap as shown in **b**. **b** Cross section of EP surface for fixed nonlinearity *α*; axis scaled with scale factor $s=2(\sqrt{1+{\alpha }^{2}/{\gamma }^{2}}-1)$. Here, ${\beta }^{{\prime} }=\beta -\gamma$. Small *α* ≪ *γ* (vicinity of linear EP, Eq. (6)): deltoid or three-cusped hypocycloid^80,82,102^ of the elliptic umbilic surface with exact scaling *s* = *α*^2^/*γ*^2^. Large *α* (farther away from linear EP, Eq. (5)): exact scaling is lost, but three-cusped shape remains. The number of eigenvectors (EVs) inside and outside the cone is indicated. The half-infinite line where parity-time (PT) symmetry is unbroken is indicated by the blue line.

The singularity set, plotted in Fig. 2a, consists of a pair of two conical surfaces with deltoid cross sections, the apices of which meet at the linear EPs at $(\alpha,{\beta }^{{\prime} },\delta )=(0,0,0)$ and (0, − 2*γ*, 0). There are four and two critical points in state variable space at each control parameter point inside and outside the cones, respectively; also see Fig. 2b. The ribs of each cone are cusp points where three critical points inside the cone meet and emerge as one critical point outside the cone. Each point on the surface between two ribs is a fold point where two critical points inside the cone meet and annihilate each other, thus vanishing on the outside. This arrangement of the fold and cusp singularities is characteristic of the elliptic umbilic catastrophe (*D*_−4_). We call the meeting point of the cones the ‘organizing point’ of the catastrophe. We show in Fig. 2b that the cone-like topological structure is not limited to the immediate neighborhood of the linear EP, but remains valid even at large nonlinearities (large *α*). More information on the EP set and the Lyapunov potential is given in Supplementary Note 4 and 5 of the Supplementary Information.

Interestingly, we see that the non-trivial topology of the set of EPs in the nonlinear parameters space (the parameter space that includes the nonlinearity *α*, as shown in Fig. 2a), is already fully determined by the EPs in the linear space (the subspace of parameters where the nonlinearity is zero).

In other words, there are infinitely many possibilities for the EP topology in the nonlinear case (cf. Fig. 1b), but already from the properties of the linear EP alone we find that the topology must be the cone-like structure. At small nonlinearities, this structure is of the form of an elliptic umbilic, a cone with a deltoid cross section (and the cone-like structure can persist at larger nonlinearities, as in our example, preserving the topology of the elliptic umbilic but not its precise geometry). This is a remarkable statement, since usually one would try to find the set of EPs in the nonlinear parameter space by searching or scanning the part of the parameter space where the nonlinearity is nonzero. To be more specific, if in Eq. (6) we set *α* = 0, ${\beta }^{{\prime} }=0$ and *δ* = 0, we are left with the so-called ‘germ’ (sometimes called degenerate core) of the catastrophe. For catastrophes with codimension up to 5 (called elementary catastrophes), the germs can be found in textbooks, for example ref. ^33,81^ and are reproduced in Supplementary Table 1. Each germ gives an unambiguous assignment of the corresponding elementary catastrophe. Since, in our case, the Lyapunov potential (as a polynomial in the state variables given by the eigenvectors) that generates the eigenvector equations of the nonlinear eigenvalue problem exists, we can predict the topology of the EP singularity set in the full parameter space solely from the Lyapunov potential where all parameters (including the nonlinearity) have been set to zero. The precise geometric shape can change under smooth transformations of parameters, but its topological properties are determined by the universal unfolding (which is defined as the versal unfolding of minimal dimension, see, e.g., p. 621 of ref. ^81^) of the elliptic umbilic singularity.

Regarding PT symmetry^35–44^, we note that the Hamiltonian in Eq. (2) is PT-symmetric, in other words [*H*, *PT*] = 0, with parity represented by the *σ*_1_ Pauli matrix, if *δ* = 0. In that case, PT symmetry is unbroken (the eigenenergies are real and the eigenvectors are common to *H* and *PT*) if $| \beta | > \sqrt{{\alpha }^{2}+{\gamma }^{2}}$. This region is indicated as thick blue line in Fig. 2b. In Fig. 2a, it is the part of the *δ* = 0 plane outside of the deltoid cone.

**Fig. 3:**
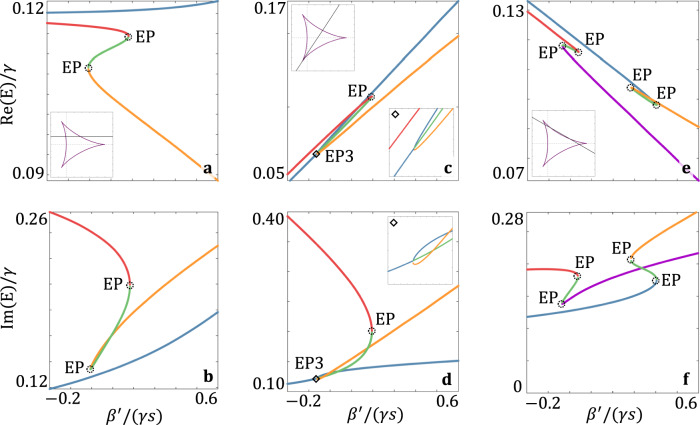
Selected complex eigenvalue traces. Complex eigenvalues (real and imaginary parts) of *H* in Eq. (2) at fixed, nonzero *α*/*γ* = 1 across lines in the $({\beta }^{{\prime} },\delta )$ plane. The insets not marked by diamonds show the $({\beta }^{{\prime} },\delta )$ plane and the line along which the spectra shown in the main parts of the figures are evaluated. Regardless of the lines' directions, we have two and four eigenvalues outside and inside the cone, respectively, in agreement with the number of values of critical points of the Lyapunov potential (see Supplementary Fig. 3 for examples of the potential landscape). At the smooth parts of the deltoid boundary (fold lines), two states are created/annihilated, and two states are unaffected by the boundary (**a**,**b**,**e**,**f**). Crossing the cusp, (**c**, **d**), three states coalesce at a third-order nonlinear exceptional point (EP), denoted as EP3, and one state is unaffected. The insets marked by diamonds show a zoomed-in plot of the eigenvalue traces close to the EP3.

The power of using catastrophe theory in the context of EPs can be seen from the following considerations. First, the information on the geometric structure of the nonlinear EPs provides immediate knowledge of the number of eigenvalues as a function of the parameters used. The potential *V*_*EU*_ has four critical points inside the cone and two outside the cone; exemplary plots of *V*_*EU*_ are shown in Supplementary Fig. 3 of the Supplementary Information. This implies that we have four eigenvalues inside the cone and two outside, and the number changes at the folds and cusps, which represent the location of the EPs. This is illustrated in Fig. 3, which shows the eigenvalues of *H* along the straight lines in ($\delta,{\beta }^{{\prime} }$)-parameter space as indicated in each row of subplots. At the apices of the cones that are shown in Fig. 2a, the four critical points coalesce, showing that, depending on how the limit is performed, the linear EP can be viewed as the limiting case of a fourth-order nonlinear EP. A stability analysis of the solutions is planned as future work. We also note that *F* might be a function of both *w* and *θ*, and the eigenvector equations may not be derivable from a Lyapunov potential. General bifurcation theory may be needed to study the conditions under which we still have an elliptic umbilic singularity.

### Extension beyond quadratic nonlinearities

In the previous subsection we discussed the example of a Kerr-like nonlinearity. We note, however, that the power of catastrophe theory lies in the fact that the linear matrix already fixes the elliptic umbilic catastrophe, and a broad class of matrices extending our case given in Eq. (2) will have the same topological singularity structure. Let us first note that the matrix in Eq. (2) is equivalent, as far as the topology of the EP object in parameter space is concerned, to one that contains self coupling (*α*_*c*_) and cross coupling (*α*_*x*_) terms with quadratic nonlinearities:

7 $${H}_{1}=\left(\begin{array}{cc}\delta+i\gamma+{\alpha }_{c}| x{| }^{2}+{\alpha }_{x}| y{| }^{2} & \beta \\ \beta & -\delta -i\gamma+{\alpha }_{c}| y{| }^{2}+{\alpha }_{x}| x{| }^{2}\end{array}\right).$$

Here, the parameter *α* used above corresponds to *α* = (*α*_*c*_ − *α*_*x*_)/2. The two matrices differ by a multiple of the unit matrix, and thus have the same eigenvectors while the eigenvalues differ only by a constant shift $\overline{\alpha }=({\alpha }_{c}+{\alpha }_{x})/2$. Here, we assume the couplings *α*_*c*_ and *α*_*x*_ to be real-valued, but below we will see that they also can be imaginary (which in some applications can be related to gain saturation) or complex.

We can generalize the matrix in Eq. (7) to allow for sufficiently smooth real-valued nonlinearity functions *f*(*u*) (for which conditions will be given below) and with complex nonlinearity parameters *α*_*c*_, *α*_*x*_

8 $${H}_{1}=\left(\begin{array}{cc}\delta+i\gamma+{\alpha }_{c}f(| x| )+{\alpha }_{x}f(| y| ) & \beta \\ \beta & -\delta -i\gamma +{\alpha }_{x}f(| x| )+{\alpha }_{c}f(| y| )\end{array}\right).$$

Similar to the case discussed above, we can subtract a diagonal matrix that is a unit matrix multiplied by the (nonlinear) constant $g({{\bf{x}}})=\overline{\alpha }(f(| x| )+f(| y| ))$, that is we define ${H}_{1}=g({{\bf{x}}})\widehat{1}+H$, where $\widehat{1}$ is the unit matrix, and obtain

9 $$H=\left(\begin{array}{cc}\delta+i\gamma+\alpha \left(\right.f(| x| )-f(| y| ) & \beta \\ \beta & -\delta -i\gamma -\alpha (f(| x| )-f(| y| ))\end{array}\right).$$

Once the nonlinear eigenvalue problem of *H* has been solved and the eigenvalues *λ*_*n*_ and eigenvectors **x**_*n*_ are known, we evaluate the constant *g*(**x**) with the eigenvectors **x**_*n*_ and obtain the eigenvalues of *H*_1_ in Eq. (8) as ${\lambda }_{n}^{(1)}$ = *λ*_*n*_ + *g*(**x**_*n*_), while the eigenvectors for *H*_1_ and *H* are the same. As in the previous section, we write the eigenvector elements as functions of *w* and *θ*, and note that the moduli of both components, $| x|=1/\sqrt{1+{w}^{2}}$ and $| y|=w/\sqrt{1+{w}^{2}}$, only depend on the real variable *w*. Hence the nonlinear term can be written as

10 $$\alpha \left(\right.f(| x| )-f(| y| )=\alpha F(w)$$

with

11 $$F(w)=f\left(\frac{1}{\sqrt{1+{w}^{2}}}\right)-f\left(\frac{w}{\sqrt{1+{w}^{2}}}\right)$$

Similar to the example of the quadratic nonlinearity discussed in the previous section, the linear EP is at *w* = 1. As mentioned at the beginning of the Results section, the condition for the elliptic umbilic singularity is that at the linear EP (where *w* = 1 in the case of symmetric matrices), the function *F*(*w*) must be approximated by a power expansion. Therefore, important examples of functions with elliptic umbilic singularity sets include all power functions *f*(*u*) = *u*^*n*^ with nonzero exponent, *n* ≠ 0, such as the quartic nonlinearity *f*(*u*) = *u*^4^, and periodic functions, such as $f(u)=\cos ({u}^{2})$.

### Different Taylor expansions

So far, we have limited the analysis to symmetric matrices where, at the EP (that is for *w* = 1) the Taylor expansion of the nonlinearity function *F*(*w*) yields *F*(1) = 0 and ${F}^{{\prime} }(1)\ne 0$. We note, however, that the theory remains valid for any real-valued function *F*(*w*) that can be approximated by a Taylor expansion in the vicinity of the EP. In this context, we consider separately four categories of functions that yield elliptic umbilic EP structures that include and extend the example discussed in Sec. 2. and 2.2. These are (1) *F*(1) ≠ 0 and ${F}^{{\prime} }(1)\ne 0$, (2) *F*(1) = 0 and ${F}^{{\prime} }(1)\ne 0$, (3) *F*(1) ≠ 0 and ${F}^{{\prime} }(1)=0$, and (4) *F*(1) = 0 and ${F}^{{\prime} }(1)=0$. Here category (2) is the one discussed above. We note that in the four categories the elliptic umbilic singularity sets emerge as functions of different parameters/variables. But common to these categories is the elliptic umbilic topology, in other words their singularity sets are topologically equivalent. Further details about the Lyapunov functions for the four categories are given in Supplementary Note 1, Sec 1.B.1, of the Supplementary Information. Examples of matrices belonging to category (1) include the Hamiltonian in Eq. (1) of ref. ^66^, given for coupled electric resonators, evaluated without noise, and Eq. (5) of ref. ^34^, with applicability for example to ring or micropillar optical resonators.

### Asymmetric matrices, complex nonlinear coupling constants

We have also considered the case of asymmetric matrices, with the off-diagonal parameters *b* and *c* in the Hamiltonian Eq. (1) being independent complex parameters. Our findings are summarized here, and the algebraic derivation is detailed in Supplementary Note 1, Secs. 1.B.3 and 1.B.4, in the Supplementary Information. As in the symmetric-matrix case, we construct a Lyapunov potential for the eigenvector elements of this asymmetric Hamiltonian in Supplementary Note 1, Sec. 1.B.3. A straightforward expansion around the linear EP shows that the germ (the limiting Lyapunov potential as a function of (*r*, *ϕ*) when the parameters are set at the EP values) formally appears as the sum of two elliptic umbilics. We derive in Supplementary Note 1, Sec. 1.B.4, a coordinate transformation that maps this structure into a canonical elliptic umbilic in the new coordinates. This shows that the (linear and nonlinear) EP sets also possess the topological structure of the elliptic.

We find that the elliptic umbilic topology is also retained when the nonlinear coupling constant *α* is imaginary or complex. We comment briefly on this case in Supplementary Note 1, Sec. 1.B.2, in the Supplementary Information.

### Symmetry in nonlinear coefficient *α*

We also mention again that the EP structure shown in Fig. 2a is symmetric with respect to the Kerr-like nonlinearity *α* and the linear coupling *β* (in other words invariant under *α* → − *α*, *β* → − *β*). For real-valued self (*α*_*c*_) and cross (*α*_*x*_) couplings in Eq. (7), it is instructive to compare our nonlinear 2 × 2 eigenvalue problem with the nonlinear Schrödinger equation (NLS). If the NLS is derived from the Helmholtz equation in paraxial approximation, then the spatial propagation coordinate *z* plays the role of time and the diffraction (second-order derivative in the transverse direction) plays the role of the kinetic energy, see e.g. ref. ^91^. While the general solution structure of the NLS includes effects like soliton formation and is generally complicated (and the NSL is not integrable in more than one spatial dimensions), it has been shown in ref. ^92^ that coupled-mode analysis in the nonlinear regime can be done with the NLS under suitable conditions. If one would use two modes with different transverse extensions, effects analogous to self-focusing and self-defocusing could be described by a 2-mode model. In the language of our eigenvalue equation, the eigenvalue *E* would correspond to the wave vector in the propagation direction *z*, and the coupling coefficients *α*_*c*_ and *α*_*x*_ would depend on the spatial overlap between the two modes. More detailed studies of the discrete NLS (also called discrete self-trapping equation) are provided in ref. ^93^, but most studies of the discrete NLS, including 2-mode models that are equivalent to the (nonlinear) pendulum^94^, do not include decay, which is essential in our study of the EP structure. If, however, a self-focusing and self-defocusing system with decay can be described by our 2-mode model, Eq. (7), with the difference in the absorption coefficients of the two modes given by *γ*, then we predict that the EP structure is symmetric in $\alpha=\frac{1}{2}({\alpha }_{c}-{\alpha }_{x})$ as well as the linear coupling *β* and the energy splitting *δ*, as shown in Fig. 2a.

### Larger matrix dimensions

For *N* × *N* matrices with *N* > 2, the dimension of the state variable space increases with *N*, the set of linear EPs is expected to have more structure than that of the *N* = 2 case. More complicated, higher-order singularities, e.g. parabolic umbilics or singularities outside of the elementary catastrophe set, may be involved, most of which have higher codimensions (minimum dimension of the parameter space to capture the full structure of the singularity set) than the elliptic umbilic. But even when a relatively complete analysis comparable to the 2 × 2 case cannot be done, using the tools of catastrophe theory and bifurcation theory may still provide fruitful insight into the topological/geometric structure of the singularity set. We also note that in higher dimensional systems that can, for example, systematically be realized and explored with coupled ring resonators^10,75^ (described by *N* × *N* matrices with *N* > 2), even in the linear case we can have EPs of various orders, for example a 3 × 3 matrix can have second and third-order linear EPs. The structure of the nonlinear EPs can then be expected to be more complex than the case of nonlinear EPs discussed above for the 2 × 2 matrix. We found that in the 2 × 2 case where a Lyapunov potential exists, the coalescence of eigenvectors agrees with the condition of singularities of the Lyapunov function, and - as is already known^65–67^ - the order of the nonlinear EPs can be higher than that of the linear EPs (in our case, the linear problem can only have second order EPs while the nonlinear problem has up to fourth-order nonlinear EPs).

## Discussion

In summary, we have shown that the rich interplay of nonlinearity and non-Hermiticity at EPs results in a non-trivial topological structure of the EPs in the space of parameters including the nonlinearity parameter *α*. This structure (or singularity set) is called elliptic umbilic and shown in Fig. 2a. Our findings apply to a large class of nonlinear systems that include—but are not limited to—all power-law nonlinearity functions *f*(*u*) in the effective non-Hermitian Hamiltonian Eq. (8) with self-coupling and cross-coupling, where the coupling coefficients can be real (in optics related to nonlinear refractive index coupling), imaginary (often related to nonlinear decay or gain), or complex. Asymmetric matrices with complex parameters in the off-diagonal elements also belong to this class. To the best of our knowledge, the class of matrices covered by our theory includes most, if not all, nonlinear EP systems that are presently discussed in the literature (Kerr-type nonlinearity, atomic and polariton condensates, superfluids, and coupled ring or micropillar optical and electric resonators). Further extensions of the class of matrices with the elliptic umbilic topological structure may be possible.

Specifically, we find that, by mapping EPs to degenerate critical points of a potential, catastrophe theory predicts that a second-order linear EP unfolds into the elliptic umbilic form. In practical terms, the EP perturbed by nonlinearity will spawn two touching cones of EPs with tri-cusped boundaries, independent of the underlying physical model. Interestingly, the non-trivial topological properties of the set of EPs in the space of parameters including the nonlinearity parameter *α* can be predicted solely from the Lyapunov potential at the linear EP (where *α* = 0). This is a strong statement made possible by catastrophe theory, and in this sense, the topology can be regarded as ‘universal’. From this topological viewpoint, the original linear EP acts as the organizing center of a higher-order singularity: a second-order EP becomes effectively fourth-order in the extended space of nonlinear parameters. These conclusions are the same for all physical platforms, e.g., photonic, atomic, and condensed-matter platforms. Our approach can be extended to the case of third (or higher) order linear EPs (e.g., refs. ^43,95–97^) perturbed by nonlinearities. Since, in the linear case, we find the germ to be that of an elliptic umbilic catastrophe, we conjecture that all nonlinearities that are real or complex functions of the variable *w* (and possibly both *w* and *θ*) defining the eigenvectors, and that are analytic in a neighborhood around the linear EPs and fulfill the relaxed vanishing-curl condition (see discussion following Eq. (16) in Methods), will lead to locally elliptic umbilic singularity sets.

Beyond the conceptual advance, our results have practical implications. Knowing the canonical cone–and–cusp shape of nonlinear EPs provides a clear blueprint for engineering non-Hermitian devices. For example, one can predict how tuning parameters (gain saturation, coupling strengths, etc.) will move or create EPs in polariton systems and lasers^98^, ring or micropillar optical resonators^10,34,75^, or coupled electric resonators (including PT-symmetric circuits)^66^. Crucially, because EP-based sensors and mode-control schemes depend on the topology of degeneracies, this universality will make it possible to establish rigorous bounds on the enhancement of sensitivity of EPs in nonlinear systems and enables the design of robust, high-sensitivity devices across different platforms. Our prediction of the singularity structure of EPs in the nonlinear regime should enable systematic studies and possible optimization of the renormalization of EPs due to noise and stochastic processes, e.g. refs. ^34,57,66,97^, as well as studies of Liouvillian EPs^99,100^. From a broader perspective, identifying a common “fingerprint” for EPs highlights a deep unity in non-Hermitian physics. We expect these principles to guide future explorations – for instance in higher-dimensional multi-mode or many-body setups – and to inform the development of new spectroscopic and sensing techniques grounded in the universal topology of EPs.

## Methods

The methodology of our theoretical approach is based on a branch of bifurcation theory called catastrophe theory. Here, we summarize the main aspects used in this method of analyzing nonlinear systems. More details are given in Supplementary Note 2 of the Supplementary Information.

We begin with a brief review of catastrophe theory. Consider a smooth function (Lyapunov potential) ${V}_{L}({x}_{1},{x}_{2},\ldots,{x}_{n},{a}_{1},{a}_{2},\ldots,{a}_{m})$ of *n* state variables *x*_*i*_, *i* = 1, …, *n* and *m* control parameters *a*_*j*_, *j* = 1, …, *m*. The critical points of *V* are points in state space where (solutions in state space to the equations)

12 $$\frac{\partial {V}_{L}}{\partial {x}_{i}}=0\,,\,i=1,\ldots,n.$$

Catastrophe theory seeks to classify the qualitative behavior of the critical points when the parameters are varied. If we think of the parameter space as a ‘phase diagram’, qualitative changes of critical point behavior happen at parameter space points for which *V* has degenerate critical points (singularities). At these points (in parameter and state spaces), the Hessian matrix of second derivatives is singular,

13 $${\rm det}\left[\frac{{\partial }^{2}{V}_{L}}{\partial {x}_{i}\partial {x}_{j}}\right]=0,$$

and multiple critical points coincide. We call the set of points in parameter space where *V*_*L*_ has degenerate critical points the singularity set. The theory classifies the structure of the singularity set locally around ‘organizing points’. Simple structures include folds, cusps, and umbilics. Complete tables for the elementary catastrophes with codimension (defined below) up to 5 were given in refs. ^80,81^. The table is reproduced as Supplementary Tab. 1 in the Supplementary Information. For each catastrophe in the table, *x* ≡ *x*_1_, *y* ≡ *x*_2_ are state variables, and the other lower-case letters in the Perturbation column are parameters. For a given parameterized function *V*_*L*_, an isolated point in the singularity set is fixed as the organizing point (in parameter space). At this parameter value, *V*_*L*_ as a function of the state variables has one or more degenerate critical points, one of which is chosen for consideration. The state coordinates are redefined with the origin shifted to this degenerate critical point, and the value of *V*_*L*_ is also shifted to zero at this point. To match a particular catastrophe, one may Taylor expand *V*_*L*_(*x*, *y*), with parameters set at the organizing point values, around the origin and compare the lowest-order terms with the standard forms in the Germ column in Supplementary Tab. 1. A smooth coordinate transformation may be needed to match the shown standard form. The singularity set, when restricted to the organizing point, may be structurally unstable: the geometric structure of the singularity set, and other properties, may change under small perturbations. The Perturbation column in Supplementary Tab. 1 shows some standard forms of perturbations that stabilize the singularity set: its geometric structure does not change under further small perturbations. The parameters equal zero at the organizing point. For each catastrophe, the number of parameters in the shown perturbation is the minimum number that can affect the stabilization. This minimum number is the codimension of the catastrophe. A more mathematically precise explanation of catastrophe theory terminology and concepts, such as germs and codimension, can be found in, for example, ref. ^101^.

Next, we discuss EPs of the (nonlinear) eigenvalue problem and the relation to catastrophe theory. Consider an eigenvalue problem with an *N* × *N* matrix which is nonlinear in the eigenvector. Suppose the eigenvector is defined by *n* real variables (a complex variable is considered as a pair of real variables), with *n*≤2*N*. Call these variables *x*_*i*_, *i* = 1, …, *n*. Suppose also that the matrix depends on *m* parameters *a*_*j*_, *j* = 1, …, *m* and the eigenvector equation, after elimination of the eigenvalue, can be written as a set of *n* equations

14 $${f}_{k}({x}_{1},\ldots,{x}_{n},{a}_{1},\ldots,{a}_{m})=0\,,\,k=1,\ldots,n$$

For each fixed parameter set $({a}_{j},\, j=1,\ldots,m)$, the solution $({x}_{i},\, i=1,\ldots,n)$ to Eq. (14) gives the eigenvector. The EPs are points in parameter space for which multiple eigenvectors, or equivalently, multiple solutions $({x}_{i},i=1,\ldots,n)$ to Eq. (14), coalesce.

If a potential function ${V}_{L}({x}_{1},{x}_{2},\ldots,{x}_{n},{a}_{1},{a}_{2},\ldots,{a}_{m})$ such that

15 $${f}_{k}({x}_{1},\ldots,{x}_{n},{a}_{1},\ldots,{a}_{m})=\frac{\partial {V}_{L}}{\partial {x}_{k}}\,,\,k=1,\ldots,n$$

can be constructed, then the nonlinear eigenvalue problem can be ‘mapped’ onto the catastrophe theory formalism. The solutions $({x}_{i},i=1,\ldots,n)$ to Eq. (14) are the critical points of *V*_*L*_, and the machinery of catastrophe theory can be used to analyze the structure of the set of EPs, which is just the singularity set (parameter space subset where *V*_*L*_ carries degenerate critical points). In our application, the linear EP is chosen as the organizing point (see previous subsection) and structurally stabilizing perturbations are identified.

A necessary condition for the potential *V*_*L*_ to exist is that the set *f*_*k*_, *k* = 1, …, *n* have ‘vanishing curl’:

16 $$\frac{\partial {f}_{i}}{\partial {x}_{j}}-\frac{\partial {f}_{j}}{\partial {x}_{i}}=0\,,\,i,j=1,\ldots,n$$

But for our purpose here, this condition can be slightly relaxed. Even if the *f*_*k*_ in question does not satisfy Eq. (16), if a related set of functions, ${f}_{k}^{{\prime} }({x}_{1},\ldots,{x}_{n},{a}_{1},\ldots,{a}_{m})$, *k* = 1, …, *n*, has the same solutions when used in Eq. (14) and is the gradient of a potential *V*_*L*_, then the critical-point analysis of *V*_*L*_ is applicable to the EPs of *f*_*k*_. This is the case for the eigenvector equations considered in this paper. The left hand sides of the real and imaginary parts of the eigenvector equations are equal to *w*∂*V*_*L*_/∂*w* and  − ∂*V*_*L*_/∂*θ* respectively.

## Supplementary information

Supplementary Information

Transparent Peer Review file

## Data Availability

The data for the main figures of this paper, Fig. 2a and b, and 3, have been deposited in the GitHub database under accession code 10.5281/zenodo.17589377

## Code availability

The Matlab code that generated the surface of the main figure (Fig. 2a), cross sections of the surface (Fig. 2b), and complex eigenvalue trajectories (Fig. 3) have been deposited in the GitHub database under accession code 10.5281/zenodo.17589377
