# Supplementary material for: Universal topology of exceptional points in nonlinear non-Hermitian systems
Source: Nat Commun. 2026 May 20;17:7051. doi: 10.1038/s41467-026-72854-2 (PMC13392374; doi:10.1038/s41467-026-72854-2)
Supplement: Supplementary file 1 — Supplementary Information [file 41467_2026_72854_MOESM1_ESM.pdf]

Supplementary Information:
Universal Topology of Exceptional Points in Nonlinear Non-Hermitian Systems

N. H. Kwong,[1] Jan Wingenbach,[2,3] Laura Ares,[2,3] Jan Sperling,[2,3] Xuekai Ma,[2] Stefan Schumacher,[1,2,3] and R. Binder[1,4]

[1] *Wyant College of Optical Sciences, University of Arizona, Tucson, AZ 85721*
[2] *Department of Physics and Center for Optoelectronics and Photonics Paderborn (CeOPP), Paderborn University, 33098 Paderborn, Germany*
[3] *Institute for Photonic Quantum Systems (PhoQS), Paderborn University, 33098 Paderborn, Germany*
[4] *Department of Physics, University of Arizona, Tucson, AZ 85721*

(Dated: April 18, 2026)

In this Supplementary Information we provide additional information on the class of matrices represented by the Hamiltonian H of the main text and broader classes of Hamiltonians (Sec. Supplementary Note 1), the theoretical basis used in the main text (Sec. Supplementary Note 2), how to obtain the surface plots of the extended elliptic umbilic catastrophe shown in Fig. 2a of the main text (Sec. Supplementary Note 3), the approximated scaling in the strongly nonlinear limit (Sec. Supplementary Note 4), and we show exemplary landscapes of the elliptic umbilic potential (Sec. Supplementary Note 5).

Supplementary Note 1. CLASS OF MATRICES WITH ELLIPTIC UMBILIC EP NEIGHBORHOOD

A. Class of matrices represented by H in main text

In this section, we motivate the specific form of the Hamiltonian H in Eq. (1) of the main text and define the class of matrices it represents. We begin with the linear case and a general (linear) 2×2 matrix, written as

$$M_0 = \begin{pmatrix} m_{11} & m_{12} \\ m_{21} & m_{22} \end{pmatrix} , \tag{1}$$

where all matrix elements are complex. Using $\bar{m} = (m_{11} + m_{22})/2$ and $D = (m_{11} - m_{22})/2$, we have

$$M_0 = \bar{m}I + \begin{pmatrix} D & m_{12} \\ m_{21} & -D \end{pmatrix} = \bar{m}I + M_1 , \tag{2}$$

with I being the identity matrix. If $\mathbf{x}$ is an eigenvector of M_1 with eigenvalue λ, then it is also an eigenvector of M_0 with eigenvalue $\lambda + \bar{m}$. The eigenvalues of M_1 are

$$\lambda_\pm = \pm\sqrt{D^2 + m_{12}m_{21}} . \tag{3}$$

The condition for two-fold degeneracy is that either both terms under the square root are separately zero, which includes the case of conventional (diabolic) degeneracies that we do not include on our analysis, or $D^2 = -m_{12}m_{21}$ with $D \neq 0$. We assume in the following all three entries to be non-zero and treat the matrices with zero entries as limiting cases. Writing $D = |D|e^{i\theta_D}$, $m_{12} = |m_{12}|e^{i\theta_{12}}$ and $m_{21} = |m_{21}|e^{i\theta_{21}}$, the condition for the EP becomes

$$|D|^2 = |m_{12}||m_{21}| , \tag{4}$$
$$\Delta\theta \equiv \theta_{12} + \theta_{21} - 2\theta_D = \pm\pi . \tag{5}$$

In the example of H used in the main text, we restricted ourselves to symmetric matrices with real off-diagonal elements, that is $m_{21} = m_{12} \in \mathbb{R}$. Extension to the case where the off-diagonal elements are complex conjugate of each other ($\theta_{21} = -\theta_{12}$) is trivial since the phase can be passed on to the eigenvector by a unitary transformation. For symmetric matrices with complex off-diagonal elements ($|m_{21}| = |m_{12}|$, $\theta_{21} = \theta_{12}$), we write

$$M_1 = e^{i\theta_{12}} M_2 , \tag{6}$$

with

$$M_2 = \begin{pmatrix} De^{-i\theta_{12}} & |m_{12}| \\ |m_{12}| & -De^{-i\theta_{12}} \end{pmatrix} \equiv \begin{pmatrix} \delta + i\gamma & \beta \\ \beta & -\delta - i\gamma \end{pmatrix} = H_0 , \tag{7}$$

with β real-valued. Here, H_0 is the Hamiltonian in Eq. (1) of the main text for the case without nonlinearity, $\alpha = 0$. If $\mathbf{x}$ is an eigenvector of M_2 with eigenvalue λ, then it is also an eigenvector of M_1 with eigenvalue $\lambda e^{i\theta_{12}}$.

To summarize the linear eigenvalue problem, we restrict ourselves to complex symmetric matrices with non-zero diagonal and off-diagonal matrix elements. If $\mathbf{x} = (x, y)$ is an eigenvector of the linear Hamiltonian H_0 with eigenvalue λ, then it is also an eigenvector of the general complex symmetric matrix M_0 with eigenvalue $\lambda e^{i\theta_{12}} + \bar{m}$.

We now discuss the extension to nonlinear matrices. We allow for diagonal nonlinearities that are Kerr-like or quadratic in the eigenvector components (representing, for example, cubic or $\chi^{(3)}$ nonlinearities in nonlinear optics), given by self and cross coupling represented by the non-linear coefficients α_c and α_x, respectively, with

$$H_1 = \begin{pmatrix} \delta + i\gamma + \alpha_c|x|^2 + \alpha_x|y|^2 & \beta \\ \beta & -\delta - i\gamma + \alpha_c|y|^2 + \alpha_x|x|^2 \end{pmatrix} . \tag{8}$$

Using the normalization of the eigenvector, $|x|^2 + |y|^2 = 1$, we have

$$H_1 = \frac{1}{2}(\alpha_c + \alpha_x)I + H \tag{9}$$

with $\alpha \equiv (\alpha_c - \alpha_x)/2$ and

$$H = \begin{pmatrix} \delta + i\gamma + \alpha(|x|^2 - |y|^2) & \beta \\ \beta & -\delta - i\gamma - \alpha(|x|^2 - |y|^2) \end{pmatrix} . \tag{10}$$

If $\mathbf{x}$ is an eigenvector of the nonlinear Hamiltonian H with eigenvalue λ, then it is also an eigenvector of the nonlinear Hamiltonian H_1 with eigenvalue $\lambda + \frac{1}{2}(\alpha_c + \alpha_x)$. Here, H is the nonlinear Hamiltonian in Eq. (1) of the main text.

In the nonlinear case the absolute magnitude of the eigenvector carries physical information, therefore it may not be normalized to one. If the normalization is $|x|^2 + |y|^2 = \mathcal{N}^2$, with real-valued $\mathcal{N}$ not necessarily being unity, then the eigenvalue equation

$$H_1\mathbf{x} = \lambda\mathbf{x} \tag{11}$$

remains formally the same, with the same eigenvalue λ, if the following substitutions are made:

$$\begin{aligned} x &\to \tilde{x} = x/\mathcal{N} \\ y &\to \tilde{y} = x/\mathcal{N} \\ \alpha_c &\to \tilde{\alpha_c} = \alpha_c\mathcal{N}^2 \\ \alpha_x &\to \tilde{\alpha_x} = \alpha_x\mathcal{N}^2 \end{aligned}$$

We should also note that the elliptic umbilic EP structure shown in Fig. 2a of the main text would be deformed if a smooth transformation of parameters is applied. Conversely, it might happen that the analysis of a given physical or mathematical model that falls into the class of models covered by our analyses does yield and EP structure exactly as in Fig. 2a of the main text. Catastrophe theory (largely based on Thom's theorem) provides statements about the topological structure. If a given model falls in our class of models and does not yield the 'canonical' elliptic umbilic structure, catastrophe theory asserts that there exists a smooth transformation of variables that will transform the EP structure of any matrix in our class to the 'canonical' elliptic umbilic structure (in other words to the elementary catastrophe as listed in Supplementary Tab. 1).

B. Broader classes of matrices

In the following, we extend our exceptional point considerations to broader classes of 2×2 nonlinear matrices.

1. More general nonlinearities

We recall that we write the eigenvectors in the form

$$\begin{pmatrix} x \\ y \end{pmatrix} = \frac{1}{\sqrt{1 + w^2}} \begin{pmatrix} 1 \\ \tilde{w} \end{pmatrix} \tag{12}$$

with $\tilde{w} = we^{i\theta}$ and assuming here normalization to 1 (unity). In Eq. (2) and (3) in the main text the nonlinear term, for the quadratic Hamiltonian H, is $\alpha F(w)$, with $F(w) = \left(1 - w^2\right) / \left(1 + w^2\right)$. We note that in this case, the elliptic umbilic unfolding form, Eq. (6) in the main text, of the Lyapunov function around the linear EP follows from the properties $F(1) = 0$, $F'(1) \equiv dF(w)/dw|_{w=1} \neq 0$. The coefficient of the r^2 term is $\alpha F'(1)$, where $F'(1) = -1$ in this case. It is then clear that for any nonlinear function that has this analytic behavior at the linear EP, the Lyapunov function is elliptic umbilic around this point. For example, we can generalize the matrix in Eq. (8) to allow for arbitrary analytic real valued nonlinearity functions $f(u)$

$$H_1 = \begin{pmatrix} \delta + i\gamma + \alpha_c f(|x|) + \alpha_x f(|y|) & \beta \\ \beta & -\delta - i\gamma - \alpha_x f(|x|) + \alpha_c f(|y|) \end{pmatrix} . \tag{13}$$

Similar to the case discussed above, we can subtract a diagonal matrix that is a unit matrix multiplied by a (nonlinear) constant and obtain

$$H = \begin{pmatrix} \delta + i\gamma + \alpha(f(|x|) - f(|y|) & \beta \\ \beta & -\delta - i\gamma - \alpha(f(|x|) - f(|y|)) \end{pmatrix} . \tag{14}$$

As before, we write the eigenvector elements as functions of w and θ, and the nonlinear terms as

$$\alpha(f(|x|) - f(|y|) = \alpha F(w) \tag{15}$$

with

$$F(w) = f(\frac{1}{\sqrt{1+w^2}}) - f(\frac{w}{\sqrt{1+w^2}}) \tag{16}$$

Similar to the example of the quadratic nonlinearity discussed in the previous section, the EP is at $w = 1$. The property $F(1) = 0$ is trivially fulfilled for all nonlinearity functions $f(u)$. But we find that also $F'(1) \neq 0$ is fulfilled for a large class of nonlinearity functions. Importantly, this includes all power-law functions, $f(u) = u^n$ with nonzero exponent, $n \neq 0$, since in this case $F'(1) = -n2^{-n/2}$ which is not equal to zero for all $n \neq 0$. An important example in this class of nonlinearity functions would be the quartic nonlinearity $f(u) = u^4$. Another example outside the power-law functions would be the periodic function $f(u) = \cos(u^2)$, which yields $F'(1) = \sin(1/2) \neq 0$. Quite generally, there are infinitely many functional forms that are covered by our analysis, meaning that they lead to elliptic umbilic EP singularities shown in Fig. 2a of the main text.

If we assume the eigenvectors to be normalized to a real constant $\mathcal{N}$ that is in general different from 1, then $x = \frac{\mathcal{N}}{\sqrt{1+w^2}}$ and $y = \frac{\mathcal{N}w}{\sqrt{1+w^2}}$ and therefore

$$F(w) = f(\frac{\mathcal{N}}{\sqrt{1+w^2}}) - f(\frac{\mathcal{N}w}{\sqrt{1+w^2}}) \tag{17}$$

This changes the derivative of the power-law model, where $f(u) = u^n$, at the EP to $F'(1) = -\mathcal{N}^n n 2^{-n/2}$ which is not equal to 0 for any $n \neq 0$.

We further extend our consideration to any analytic real function $F(w)$ multiplied by a real coefficient α In this case, the eigenvector equations are still a set of gradient vector field equations, and a Lyapunov function exists. Explicitly, the eigenvector equations with $F(w)$ are

$$2\alpha F(w) + 2\delta + \beta \left[w - \frac{1}{w}\right] \cos\theta = 0 \tag{18}$$

$$2\gamma + \beta \left[w + \frac{1}{w}\right] \sin\theta = 0 \tag{19}$$

As components of a vector field in (w, θ) space, the left hand sides of the two equations can be expressed as $\left[w\partial V_L/\partial w, -\partial V_L/\partial\theta\right]^T$ with the Lyapunov function V_L being

$$V_L(w, \theta, \alpha, \beta, \gamma, \delta) = 2\alpha \int^w dw' \frac{F(w')}{w'} + 2\delta \ln w + \beta \left[w + \frac{1}{w}\right] \cos\theta - 2\gamma\theta \tag{20}$$

In the Hamiltonian H and its generalization discussed above, the nonlinearity function $F(w)$ has the analytic properties $F(1) = 0$, $F'(1) \equiv \left.\frac{dF(w)}{dw}\right|_{w=1} \neq 0$. These properties are not necessary conditions for the Lyapunov potential

to have an elliptic umbilic structure around the linear EP. We show below that for four types of analytic behavior at $w = 1$: (1) $F(1) \neq 0, F'(1) \neq 0$, (2) $F(1) = 0, F'(1) \neq 0$, (3) $F(1) \neq 0, F'(1) = 0$, (4) $F(1) = 0, F'(1) = 0$, the Lyapunov potential is elliptic umbilic locally. In each case, we expand the integral $\int^x \frac{F(x')}{x'} dx'$ in the Lyapunov potential Eq. (20) around $w = 1$ up to r^2 or $r^3, r = w - 1$ (and expand the other terms in the usual way). We disregard the constant term $\int^1 \frac{F(x')}{x'} dx'$.

(1) $F(1) \neq 0, F'(1) \neq 0$. The Lyapunov potential has the limiting form

$$V_L(r, \phi) \sim -\frac{\phi^3}{3} + \phi r^2 + 2\frac{\beta'}{\gamma}\phi + \frac{2}{\gamma}(\alpha F(1) + \delta)r + \frac{\alpha}{\gamma}\left[F'(1) - F(1)\right] r^2 \tag{21}$$

(2) $F(1) = 0, F'(1) \neq 0$. The limiting form is

$$V_L(r, \phi) \sim -\frac{\phi^3}{3} + \phi r^2 + 2\frac{\beta'}{\gamma}\phi + \frac{2\delta}{\gamma}r + \frac{\alpha}{\gamma}F'(1)r^2 \tag{22}$$

This case includes H in the main text and its generalizations discussed above.

(3) $F(1) \neq 0, F'(1) = 0$. The limiting form is

$$V_L(r, \phi) \sim -\frac{\phi^3}{3} + \phi r^2 + 2\frac{\beta'}{\gamma}\phi + \frac{2}{\gamma}(\alpha F(1) + \delta)r - \frac{\alpha}{\gamma}F(1)r^2 \tag{23}$$

(4) $F(1) = 0, F'(1) = 0$. Categories (2) and (3) can be seen as special cases of Category (1). Category (4) here warrants more consideration. The limiting form is

$$V_L(r, \phi) \sim -\frac{\phi^3}{3} + \phi r^2 + 2\frac{\beta'}{\gamma}\phi + \frac{2\delta}{\gamma}r + \overline{\alpha'}r^3 \tag{24}$$

where

$$\overline{\alpha'} = \frac{\alpha}{3\gamma}F''(1)$$

For the relevant unfolding, we look for terms of order less than that of the germ. In this case, they are linear or quadratic in r, ϕ. So $\overline{\alpha'}r^3$ is not relevant to the topological structure of the bifurcation set near the linear singular point. Also noted is that the functions whose coefficients are β' or δ in the full Lyapunov potential have quadratic terms in r, ϕ in general. Formally, we can also transform $-\frac{\phi^3}{3} + \phi r^2 - \bar{\alpha}' r^3$ into the standard form of $-\frac{u^3}{3} + uv^2$ by a linear coordinate transformation

$$r = au + bv$$
$$\phi = cu + dv$$

with a, b, c, d to be determined. Substitution into the third-order terms give

$$-\frac{(cu + dv)^3}{3} + (cu + dv)(au + bv)^2 - \overline{\alpha'}(cu + dv)^3$$

Expanding the terms gives four third-order terms

$$p_1 u^3 + p_2 u^2 v + p_3 uv^2 + p_4 v^3$$

where p_1, p_2, p_3 are third-order polynomials of a, b, c, d. Setting

$$p_1 = -\frac{1}{3}, p_2 = 0, p_3 = 1, p_4 = 0$$

we can solve, analytically or numerically, for the appropriate values of a, b, c, d to give $-\frac{u^3}{3} + uv^2$.

The above shows that the Lyapunov potential has an elliptic umbilic structure in a neighborhood of the linear EP if the nonlinear function $F(w)$ is analytic in that neighborhood. In the main-text model, the EU structure appears to extend to the whole three-dimensional parameter space. For $F(w)$ in general, it is expected that the EU structure is valid locally around the linear EP in parameter space. Even so, the parameter space region of EU validity is typically much larger than the region where the EU unfolding form is a good numerical approximation to V_L.

2. Imaginary nonlinearity coefficient α

A similar analysis for the case that α is purely imaginary yields a EU germ similar to the one in the case of α being purely real, but with ϕ and r interchanged. In this case, the catastrophe remains elliptic umbilic. In the case of complex α with nonzero real and imaginary part, the germ becomes a sum of two elliptic umbilic forms. This can be reduced to the canonical elliptic umbilic form through a smooth coordinate transformation as shown in Sec. Supplementary Note 1 B 4.

3. Asymmetric matrices

The Hamiltonian used in the main text and its generalizations discussed in previous subsections are symmetric. In this subsection, we discuss the extension of the method to the case of asymmetric matrices.

We consider the EP of the matrix M_1 in Eq. (2). For notational convenience, we write m_{12} as $b = |b|e^{i\theta_b}$ and m_{21} as $c = |c|e^{i\theta_c}$. The eigenvector is written in our usual form with $\tilde{w} = we^{i\theta}$. Explicitly,

$$M_1 = \begin{pmatrix} D + \alpha F(w) & b \\ c & -D - \alpha F(w) \end{pmatrix} \tag{25}$$

where $D = |D|e^{i\theta_D} = \delta + i\gamma$ is a complex parameter, α is real, and $F(w)$ is within the class of nonlinear functions discussed in the previous subsection. We first consider the linear limit. At $\alpha = 0$, the eigenvalues $\lambda_\pm$ are given by Eq. (3). The corresponding $\tilde{w}$ in the eigenvectors are

$$\tilde{w}_\pm = \frac{\pm\sqrt{bc + D^2} - D}{b} \tag{26}$$

The expressions Eqs. (3) and (26) show that the exceptional points lie on the parameter set $c = -D^2/b$, and the coalescing eigenvalue and eigenvector at each EP are $\lambda = 0$ and $\tilde{w} = -D/b$ respectively. In magnitude-phase form, these EP conditions on c and $\tilde{w}$ are

$$|c| = |D|^2/|b| \quad , \quad \theta_c = \pi + 2\theta_D - \theta_b \tag{27}$$

$$w = |D|/|b| \quad , \quad \theta = \pi + \theta_D - \theta_b \tag{28}$$

We return to the nonlinear matrix and map the eigenvalue equations to the critical point equations of a Lyapunov function. Eliminating the eigenvalue λ from the matrix equation, we get the eigenvector equation with real and imaginary parts being

$$2\alpha F(w) + 2\delta + |b|w\cos(\theta_b + \theta) - \frac{|c|}{w}\cos(\theta_c - \theta) = 0 \tag{29}$$

$$2\gamma + |b|w\sin(\theta_b + \theta) - \frac{|c|}{w}\sin(\theta_c - \theta) = 0 \tag{30}$$

The Lyapunov function is constructed as

$$V_L(w, \theta, \alpha, |b|, \theta_b, |c|, \theta_c, \gamma, \delta) = 2\alpha \int^w dw' \frac{F(w')}{w'} + 2\delta \ln w - 2\gamma\theta + |b|w\cos(\theta_b + \theta) + \frac{|c|}{w}\cos(\theta_c - \theta) \tag{31}$$

It can be verified that the left hand sides of Eqs. (29) and (30), when considered as components of a vector field in (w, θ) space, is equal to $[w\partial V_L/\partial w, -\partial V_L/\partial\theta]^T$. The eigenvector (w, θ) is a critical point of V_L, and the coalescing eigenvector at an EP is a degenerate critical point of V_L.

As in the symmetric-matrix case, we assume a linear EP to be the organizing point of the EP structure in the parameter space of the nonlinear eigenvector and seek a match with the germ of a catastrophe around the coalescing eigenvector. To this end, we set $\alpha = 0$, apply the EP condition Eq. (27) on c, and expand V_L in (w, θ) around the linear coalescing eigenvector Eq. (28). Writing $w = |D|/|b| + r$, $\theta = (\pi + \theta_D - \theta_b) + \phi$, we obtain V_L in leading order of r and ϕ as

$$V_L(w, \theta, \alpha = 0, b, c = -D^2/b, D) = V_0 + \gamma\left[-\frac{\phi^3}{3} + \left(\frac{|b|}{|D|}r\right)^2\phi\right] + \delta\left[-\frac{1}{3}\left(\frac{|b|}{|D|}r\right)^3 + \left(\frac{|b|}{|D|}r\right)\phi^2\right] + O(r^k\phi^{4-k}) \tag{32}$$

$$V_0 = -2\gamma(\pi + \theta_D - \theta_b) + 2\delta\ln\frac{|D|}{|b|} \tag{33}$$

$k=0,...,4$. V_0 does not depend on (w,θ) and is irrelevant. Compared to the symmetric-matrix case, there are two complications in the analysis of the V_L in Eq. ((32)). The first, mentioned above, is that the linear EPs make up a continuous set in parameter space instead of being isolated points. The second is that Eq. (32) is a sum of two elliptic umbilic forms. This form can be reduced to the canonical elliptic umbilic form through a smooth coordinate transformation as shown in Sec. Supplementary Note 1 B 4. The elliptic umbilic nonlinear EP surface is then normal to the tangent plane of the linear EP line, surface or hypersurface.

4. Transformation between canonical elliptic umbilic and double elliptic umbilic

In this subsection, we provide a smooth coordinate transformation from the (symmetric) double-elliptic umbilic germ to the canonlical elliptic umbilic germ. For clarity, we first show the reverse transformation. We write the canonical elliptic umbilic germ as

$$V_g = -\frac{x^3}{3} + xy^2 \tag{34}$$

We use the variable transformation

$$x = +au + bv \tag{35}$$
$$y = -bu + av \tag{36}$$

where (u,v) are the new variables and a,b are real-valued constants. Substitute this into Eq. (34) we have

$$V_g = -\frac{(au+bv)^3}{3} + (au+bv)(av-bu)^2 \tag{37}$$

After expanding the products, we obtain the following symmetric double elliptic umbilic form

$$V_g = a\left(a^2-3b^2\right)\left[-\frac{u^3}{3}+uv^2\right] + b\left(b^2-3a^2\right)\left[-\frac{v^3}{3}+vu^2\right] \tag{38}$$

This proves that there exists a smooth parameter transformation from the canonical to the symmetry double elliptic umbilic forms.

We now show that there also exists a coordinate transformation from the double elliptic umbilic to the canonical elliptic umbilic form. Suppose the germ is given in the symmetric double elliptic umbilic form

$$V_g = \xi\left[-\frac{u^3}{3}+uv^2\right] + \eta\left[-\frac{v^3}{3}+vu^2\right] \tag{39}$$

where ξ,η are given real-valued constants. To convert to the canonical form, we can work backwards from (38) and calculate the transformation coefficients a,b in terms of ξ,η. Comparing (38) and (39) gives

$$a\left(a^2-3b^2\right) = \xi \tag{40}$$
$$b\left(b^2-3a^2\right) = \eta \tag{41}$$

Equation (40) implies

$$b^2 = \frac{1}{3a}\left(a^3-\xi\right) \tag{42}$$

Substituting into (41) gives, after some manipulation,

$$\left(a^3-\xi\right)\left(8a^3+\xi\right)^2 = 27\eta^2a^3 \tag{43}$$

which is a cubic equation in a^3. Expanding the left-hand side gives

$$64a^9 - 48\xi a^6 - \left(15\xi^2+27\eta^2\right)a^3 - \xi^3 = 0 \tag{44}$$

For any given ξ,η, solving (44) gives a^3 (and hence a) and (42) gives b. Furthermore, since eq. (44) is cubic equation in a^3 with real coefficients, there exists at least one real-valued solution for all real parameters ξ and η, proving that

the double elliptic umbilic form can always be reduced to the canonical form with a smooth coordinate transformation.

5. *Summary*

We have shown that the set of exceptional points of the matrices of the form M_1 in Eq. (25) have an elliptic umbilic singularity structure around the linear EP under the following conditions. The parameter D in the diagonal can be complex, and the coefficient of the nonlinearity α can also be complex. $F(w)$ is a real analytic function of the eigenvector parameter w in the vicinity of the linear EP. The off-diagonal parameters b and c are independent complex parameters.

Supplementary Note 2. THEORETICAL BASIS: APPLYING CATASTROPHE THEORY TO EXCEPTIONAL POINTS

In this section, we provide the theoretical basis for using catastrophe theory in the context of exceptional points (EPs). While in the main text we restrict ourselves to the case of isolated second-order EPs, the general approach outlined in the following is valid for any dimension. All variables and parameters, unless designated as complex, are real.

A. Catastrophe theory

Consider a smooth function (Lyapunov potential) $V_L\left(x_1, x_2, ..., x_n, a_1, a_2, ..., a_m\right)$ of n state variables $x_i, i = 1, ..., n$ and m control parameters $a_j, j = 1, ..., m$. The critical points of V are points in state space where (solutions in state space to the equations)

$$\frac{\partial V_L}{\partial x_i} = 0 \quad , \quad i = 1, ..., n\,. \tag{45}$$

Catastrophe theory seeks to classify the qualitative behavior of the critical points when the parameters are varied. If we think of the parameter space as a 'phase diagram', qualitative changes of critical point behavior happen at parameter space points for which V has degenerate critical points (singularities). At these points (in parameter and state spaces), the Hessian matrix of second derivatives is singular,

$$\det\left[\frac{\partial^2 V_L}{\partial x_i \partial x_j}\right] = 0\,, \tag{46}$$

and multiple critical points coincide. We call the set of points in parameter space where V_L has degenerate critical points the singularity set. The theory classifies the structure of the singularity set locally around 'organizing points'. Simple structures include folds, cusps, and umbilics. Complete tables for the elementary catastrophes with codimension (defined below) up to 5 were given in Refs. [1,2], reproduced here in Supplementary Tab. 1. For each catastrophe in the table, $x \equiv x_1$, $y \equiv x_2$ are state variables, and the other lower-case letters in the Perturbation column are parameters. For a given parameterized function V_L, an isolated point in the singularity set is fixed as the organizing point (in parameter space). At this parameter value, V_L as a function of the state variables has one or more degenerate critical points, one of which is chosen for consideration. The state coordinates are redefined with the origin shifted to this degenerate critical point, and the value of V_L is also shifted to zero at this point. To match a particular catastrophe, one may Taylor expand $V_L(x, y)$, with parameters set at the organizing point values, around the origin and compare the lowest-order terms with the standard forms in the Germ column in Supplementary Tab. 1. A smooth coordinate transformation may be needed to match the shown standard form. The singularity set, when restricted to the organizing point, may be structurally unstable: the geometric structure of the singularity set, and other properties, may change under small perturbations. The Perturbation column in Supplementary Tab. 1 shows some standard forms of perturbations that stabilize the singularity set: its geometric structure does not change under further small perturbations. The parameters equal zero at the organizing point. For each catastrophe, the number of parameters in the shown perturbation is the minimum number that can effect the stabilization. This minimum number is the codimension of the catastrophe. A more mathematically precise explanation of catastrophe theory terminology and concepts, such as germs and codimension, can be found in, for example, Ref. [3[.

Supplementary Table 1: Elementary catastrophes of Thom for codimension up to $5^{(*)}$

Name	Symbol	Germ	Perturbation
Fold	A_2	x^3	ux
Cusp	$A_{\pm 3}$	$\pm x^4$	$ux^2 + vx$
Swallowtail	A_4	x^5	$ux^3 + vx^2 + wx$
Butterfly	$A_{\pm 5}$	$\pm x^6$	$tx^4 + ux^3 + vx^2 + wx$
Wigwam	A_6	x^7	$sx^5 + tx^4 + ux^3 + vx^2 + wx$
Elliptic umbilic	D_{-4}	$x^3/3 - xy^2$	$ay^2 + bx + cy$
Elliptic umbilic	D_{-4}	$x^3/3 - xy^2$	$ax^2 + bx + cy$
Elliptic umbilic	D_{-4}	$x^3/3 - xy^2$	$a(x^2 + y^2) + bx + cy$
Elliptic umbilic	D_{-4}	$x^2y - y^3$	$wy^2 + vy + ux$
Hyperbolic umbilic	D_{+4}	$x^2y + y^3$	$wy^2 + vy + ux$
Hyperbolic umbilic	D_{+4}	$x^3 + y^3$	$wxy + vy + ux$
Parabolic umbilic	D_5	$x^2y + y^4$	$ty^2 + wx^2 + vy + ux$
Second elliptic umbilic	D_{-6}	$x^2y - y^5$	$sy^3 + ty^2 + ux^2 + vy + wx$
Second hyperbolic umbilic	D_{+6}	$x^2y + y^5$	$sy^3 + ty^2 + ux^2 + vy + wx$
Symbolic umbilic	$E_{\pm 6}$	$x^3 \pm y^4$	$sxy^2 + ty^2 + uxy + vy + wx$

$^{(*)}$After Refs. [1,2]. The different versions of the umbilics are equivalent under coordinate transformation.

B. Exceptional points of the (nonlinear) eigenvalue problem

Consider an eigenvalue problem with an $N \times N$ matrix which is nonlinear in the eigenvector. Suppose the eigenvector is defined by n real variables (a complex variable is considered as a pair of real variables), with $n \leq 2N$. Call these variables $x_i, i = 1, ..., n$. Suppose also that the matrix depends on m parameters $a_j, j = 1, ..., m$ and the eigenvector equation, after elimination of the eigenvalue, can be written as a set of n equations

$$f_k\left(x_1, ..., x_n, a_1, ..., a_m\right) = 0 \quad , \quad k = 1, ..., n \tag{47}$$

For each fixed parameter set $(a_j, j = 1, ..., m)$, the solution $(x_i, i = 1, ..., n)$ to Eq. (47) gives the eigenvector. The exceptional points are points in parameter space for which multiple eigenvectors, or equivalently, multiple solutions $(x_i, i = 1, ..., n)$ to Eq. (47), coalesce.

If a potential function $V_L\left(x_1, x_2, ..., x_n, a_1, a_2, ..., a_m\right)$ such that

$$f_k\left(x_1, ..., x_n, a_1, ..., a_m\right) = \frac{\partial V_L}{\partial x_k} \quad , \quad k = 1, ..., n \tag{48}$$

can be constructed, then the nonlinear eigenvalue problem can be 'mapped' onto the catastrophe theory formalism. The solutions $(x_i, i = 1, ..., n)$ to Eq. (47) are the critical points of V_L, and the machinery of catastrophe theory can be used to analyze the structure of the set of exceptional points, which is just the singularity set (parameter space subset where V_L carries degenerate critical points). In our application, the linear EP is chosen as the organizing point (see previous subsection) and structurally stabilizing perturbations are identified.
A necessary condition for the potential V_L to exist is that the set f_k, $k = 1, ..., n$ have 'vanishing curl':

$$\frac{\partial f_i}{\partial x_j} - \frac{\partial f_j}{\partial x_i} = 0 \quad , \quad i, j = 1, ..., n \tag{49}$$

But for our purpose here, this condition can be slightly relaxed. Even if the f_k in question does not satisfy Eq. (49), if a related set of functions, $f'_k(x_1, ..., x_n, a_1, ..., a_m)$, $k = 1, ..., n$, has the same solutions when used in Eq. (47) and is the gradient of a potential V_L, then the critical-point analysis of V_L is applicable to the EPs of f_k. This is the case for the eigenvector equations considered in this paper. The left hand sides of the real and imaginary parts of the eigenvector equations are equal to $w\partial V_L/\partial w$ and $-\partial V_L/\partial \theta$ respectively.

Supplementary Note 3. GENERATING FIG. 2A OF MAIN TEXT

We write the nonlinear eigenvalue problem as

$$\begin{pmatrix} \alpha(|x|^2 - |y|^2) + \delta + i\gamma & \beta \\ \beta & -\alpha(|x|^2 - |y|^2) - \delta - i\gamma \end{pmatrix} \begin{pmatrix} x \\ y \end{pmatrix} = E \begin{pmatrix} x \\ y \end{pmatrix} \tag{50}$$

Supplementary Table 2: Range of θ

Sector	α/γ	β/γ	Range of θ	Quadrant
I	>0	>0	$[3\pi/2, \arctan(-\gamma/\alpha)+2\pi]$	4th
II	<0	>0	$[\arctan(-\gamma/\alpha)+\pi, 3\pi/2]$	3rd
III	<0	<0	$[\arctan(-\gamma/\alpha), \pi/2]$	1st
IV	>0	<0	$[\pi/2, \arctan(-\gamma/\alpha)+\pi]$	2nd

$\arctan(-\gamma/\alpha)$ values between $-\pi/2$ and $\pi/2$

with the eigenvectors

$$\begin{pmatrix} x \\ y \end{pmatrix} = \frac{1}{\sqrt{1+w^2}} \begin{pmatrix} 1 \\ \tilde{w} \end{pmatrix} \tag{51}$$

with $\tilde{w} = we^{i\theta}$. The Lyapunov potential V_L generating the eigenvector equations after eliminating E (one complex equation for $\tilde{w}$ or two real equations for w and θ) is given in the main text. Those two real equations, plus the equation $\det[h] = 0$, where h is the Hessian matrix of the second-order partial derivatives of V_L, give us three equations. After scaling the parameters by γ, we have 5 unknowns, namely $\{\alpha/\gamma, \beta/\gamma, \delta/\gamma, w, \theta\}$. In principle, one can eliminate the two state variables w and θ, which then yields an equation for a bifurcation set surface (representing coalescing eigenstates) in the 3-dimensional space of control variables. Figure 2b in the main text is obtained in a different way, adopted from Refs. [1,4,5]. One state variable, w, is eliminated, making the control variables a function of the other state variable, θ. Scanning the control parameters as a function of θ then yields the surface in the control parameter space.

The two linear EPs are at

$$(\beta/\gamma, \delta/\gamma) = (1, 0), (w, \theta) = (1, 3\pi/2) \tag{52}$$

and

$$(\beta/\gamma, \delta/\gamma) = (-1, 0), (w, \theta) = (1, \pi/2). \tag{53}$$

In the nonlinear case, $\alpha \neq 0$, the the eigenvector equations together with the vanishing Hessian equation yield the following functional form of the control parameters β and δ on θ for fixed α:

$$\frac{\beta}{\gamma} = \pm \frac{1}{\sqrt{\sin^3\theta \left(\sin\theta - \frac{\alpha}{\gamma}\cos\theta\right)}} \tag{54}$$

$$\frac{\delta_\pm}{\gamma} = \left(\frac{1}{p} - \frac{p}{1 \pm \sqrt{1-p^2}}\right)\left(\frac{\alpha}{\gamma}p + \frac{\beta}{\gamma}\cos\theta\right) \tag{55}$$

with

$$p = \frac{\beta}{\gamma}\sin\theta \tag{56}$$

and the magnitude w of the eigenvector element is given by

$$w_\pm = -\frac{1}{p} \pm \sqrt{\frac{1}{p^2} - 1} \tag{57}$$

The range of the EP θ is specified in Supplementary Tab. 2. and sketched in Supplementary Fig. 1. The exceptional point eigenvectors for fixed values of α are plotted on the complex $\tilde{w}$ plane in Supplementary Fig. 2. As shown in Supplementary Tab. 2, the parameter space is divided into four sectors according to the sign combinations of α/γ and β/γ. They correspond to the four deltoid cones in Fig. 2a of the main text. In each sector, for each value of $\alpha/\gamma, \beta/\gamma, \theta$, there are two solutions for the EP eigenvector magnitude w, and two solutions for δ, labeled by $\pm$ in Eqs. (55) and (57). There is a one-to-one correspondence between the points on the EP eigenvector curves in Supplementary Fig. 2 Sector I and the EP points on the deltoid cone sections shown in Fig. 2b of the main text for $\alpha/\gamma = 1, 10$. The three EP eigenvector values corresponding to the three cusp points are shown on one curve in Sector 1 of Supplementary Fig. 2 .

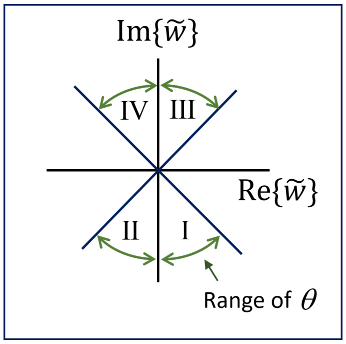

Supplementary Fig. 1: Sectors in the complex $\tilde{w}$ plane, showing the θ-ranges according to Supplementary Tab. 2.

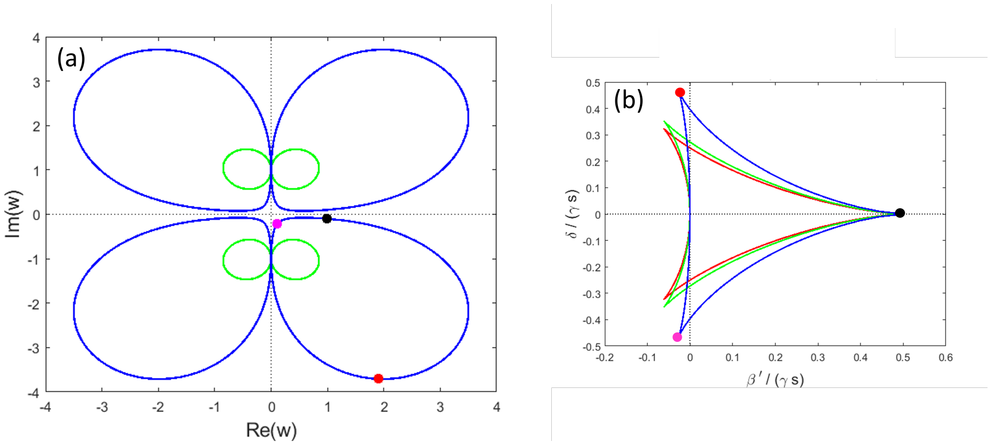

Supplementary Fig. 2: (a) Exceptional point eigenvectors (given by Eq. (57)) in the complex $\tilde{w}$ plane traced along sections of constant α/γ of the EP surface, $|\alpha/\gamma| = 1$ (green), $|\alpha/\gamma| = 10$ (blue). The two linear EP wavefunctions are at $\tilde{w} = \pm i$. The three solid circles on the blue curve in Sector I correspond to the three cusp points in (b) (same data as Fig. 2a and 2b of the main text) for $\alpha/\gamma = 10$: cusp point at $\delta = 0$ (black), $\delta > 0$ (red) and $\delta < 0$ (magenta).

Supplementary Note 4. APPROXIMATE SCALING IN STRONG NONLINEAR LIMIT

An analysis of the EP topology can be performed on the model Lyapunov function V_L, Eq. (4) of the main text which is valid in the whole parameter domain, not only very close to the linear EP. The degenerate critical points and the bifurcation set are calculated by solving the critical point equations and the Hessian determinant equation simultaneously. The bifurcation set for $\beta > 0$ is plotted in Fig. 2a of the main text and sections of it at fixed values of α/γ are plotted in Fig. 2b of the main text. The topological structure characteristic of the elliptic umbilic catastrophe is retained: the bifurcation set is made up of two triangular conical surfaces with three cusp ribs and fold surfaces between the ribs. The Lyapunov function has four (two) critical points when the control parameters are inside (outside) the cones. Algebraically, there are differences between the two bifurcation sets. While the sections of the elliptic umbilic bifurcation set scale with $s = \alpha^2/\gamma^2$, no exact scaling is found in the full model. In Fig. 2b of the main text we approximately scale the sections with the factor $s = 2\left[\sqrt{1 + \alpha^2/\gamma^2} - 1\right]$. This factor approaches quadratic scaling, α^2/γ^2 for small α but tends towards linear scaling, $2|\alpha/\gamma|$, at large α. The bifurcation set for $\beta < 0$ is a reflection image of that for $\beta > 0$ about the plane $\beta = 0$. The elliptic umbilic bifurcation sets cross this plane but those of the full model do not.

Supplementary Note 5. EXAMPLE OF LYAPUNOV POTENTIAL LANDSCAPES

In the main text, we show that the bifurcation sets are triangular (3–cusped) cones, with 4 eigenvalues inside and 2 outside. Plotting the Lyapunov potential as a function of the state variables for representative control parameters inside and outside the cone helps illustrate the relation between the number of eigenvalues and the number of critical

points (in other words points at which $\nabla V_L = 0$). Following Ref. [1], we show in Supplementary Fig. 3 representative landscapes of V_{EU}, that is the Lyapunov potential in the weakly-nonlinear limit. Inside the cone, Supplementary Fig.3a, we have four critical points (or eigenvalues), and outside, Supplementary Fig.3b, only two. The number of eigenvalues changes on the surface of the elliptic umbilic shape shown in Fig. 2a of the main text, in other words at the exceptional points. In other words, knowledge of the critical points of the Lyapunov potential is sufficient to predict the number of eigenvalues and the existence of a surface (in the control-parameter space) at which exceptional points exist and at which the number of eigenenergies changes between two and four.

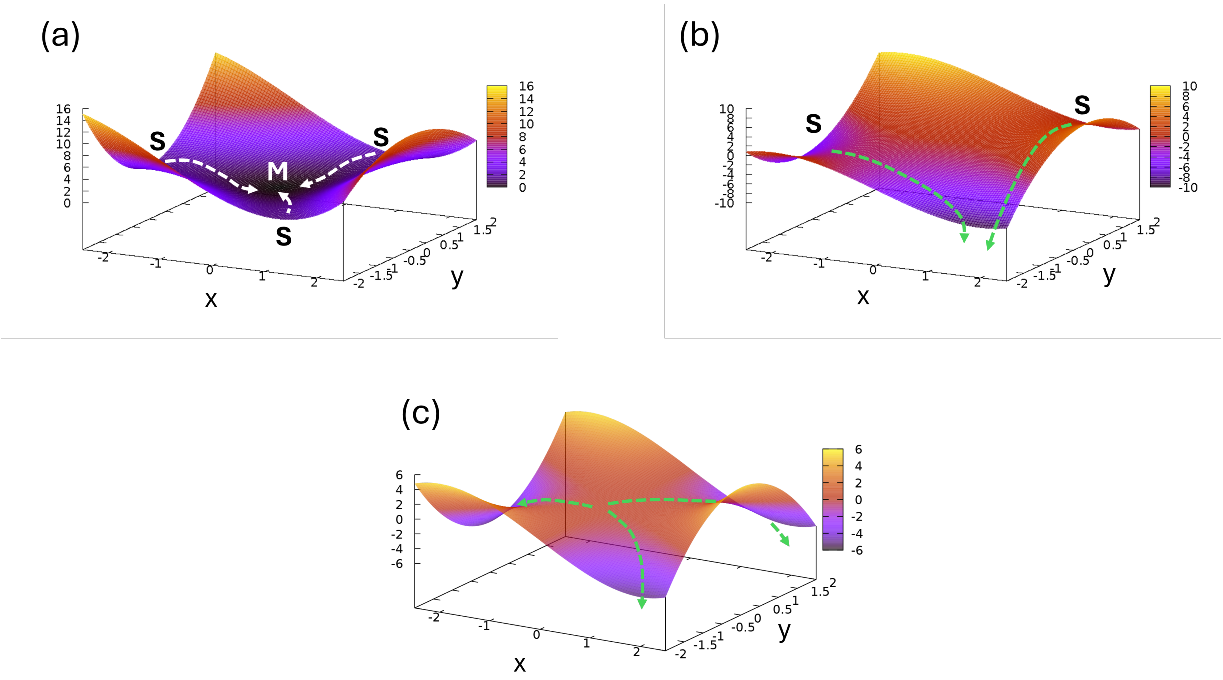

Supplementary Fig. 3: Examples of the Lyapunov function corresponding to the elliptic umbilic catastrophe $V_{EU}(x,y) = \frac{x^3}{3} - xy^2 + W(x^2+y^2) - Ux + Vy$, using the standard textbook notation (Ref. [1]) of x and y for the state variables (rather than ϕ and r used in Eq. (5) of the main text). (a) $U,V = 0$, $W = 1$ inside cone, four critical points (three saddle points 'S', one minimum 'M'), (b) $U,W = 0$, $V = 2$ outside cone, two critical points (saddle), (c) $U,V,W = 0$ 'germ' only (without unfolding), corresponding to the organizing point of the catastrophe. On the surface of the cone (exceptional points), shown in Fig. 2a of the main text, the number of solutions (eigenvalues) changes between two and four. The dashed arrows show hypothetical paths that a classical particle moving (or falling downhill) in this potential landscape might take, if it were initially at one of the critical points. In this potential, a classical particle would have a stable minimum (M) only inside the cone with $W > 0$, as seen in (a).

Supplementary References

[1] P. T. Saunders, *An introduction to catastrophe theory* (Cambridge University Press, Cambridge, 1980).
[2] R. Gilmore, *Catastrophe theory for scientists and engineers* (Wiley, New York, 1981).
[3] D. P. L. Castrigiano and S. A. Hayes, *Catastrophe Theory, 2nd ed.* (Westview Press, Boulder, 2004).
[4] T. Brocker and L. Lander, *Differentiable Germs and Catastrophes* (Cambridge University Press, London, 1975).
[5] T. Poston and I. Stewart, *Catastrophe: Theory and Its Applications* (Dover, New York, 1978).
