# Supplementary material for: Universal topology of exceptional points in nonlinear non-Hermitian systems
Source: Nat Commun. 2026 May 20;17:7051. doi: 10.1038/s41467-026-72854-2 (PMC13392374; doi:10.1038/s41467-026-72854-2)
Supplement: Supplementary file 2 — Transparent Peer Review file [file 41467_2026_72854_MOESM2_ESM.pdf]

nature portfolio

Peer Review File

# Universal topology of exceptional points in nonlinear non-Hermitian systems

Corresponding Author: Professor Rolf Binder

**This file contains all reviewer reports in order by version, followed by all author rebuttals in order by version.**

**Attachments originally included by the reviewers as part of their assessment can be found at the end of this file.**

Version 0:

Reviewer comments:

Reviewer #1

(Remarks to the Author)
The authors of the manuscript "Universal Topology of Exceptional Points in Nonlinear Non-Hermitian Systems" study the nonlinear eigenvalue problem for a class of 2x2 non-Hermitian matrices and provide some physical examples that motivate such a study. They analyze exceptional points (EP) that emerge in this setup and link them to the catastrophe theory. In particular, the authors investigate how a manifold of the EPs in the space of system's parameters is deformed due to the nonlinearity compared to the linear eigenvalue problem.

The nonlinear eigenvalue problem that the authors consider consists in finding the eigenvalues and eigenvectors of a non-Hermitian Hamiltonian, which itself explicitly depends on the components of the eigenvectors. This, effectively, reduces to a solution of a system of non-linear equations. The authors analyze a surface of EP locations in the three-dimensional space of system's parameters (a surface of points at which EPs occur). The shape of this surface is connected to a particular type of catastrophes.

For the linear eigenvalue problem, a 2x2 matrix can contain only square root EPs that correspond to the fold catastrophe. In the non-linear regime, the EPs structure can be more involved (since the characteristic equation can be a polynomial of a power higher than 3). For the class of nonlinearities considered by the authors, there exist either two or four exceptional points in different parts of the 3D parametric space, and these regimes are separated by the exceptional surface that corresponds to the elliptic umbilic catastrophe.

The connection between the EPs and the catastrophe theory is quite well-known (as well as the topological consequences of such a connection) for the linear eigenvalue problems. There are many papers on EPs that study this connection implicitly. We mention here a few examples of papers that discuss this connection explicitly: Phys. Rev. B 102, 245144 (2020), Phys. Rev. Lett. 127, 186602 (2021), Nature Phys. 19, 1098–1103 (2023), Phys. Rev. Res. 6, 043209 (2024).

The main novelty claimed by the authors is the extension of the connection between the EPs and the theory of catastrophes to the non-linear case. The manuscript contains interesting novel results beyond this extension. The authors find a catastrophe formed by the exceptional surface within the 3D space of the system's parameters (so each point on its surface corresponds to its own catastrophe), which would not be possible for a linear eigenvalue problem.

Regarding the technical details – the analysis is sound and adequate, the calculations presented in the manuscript and the Supplementary Information can be reproduced.
To summarize, the manuscript contains novel and interesting results that justify its publication. The authors generalize a known concept, but this generalization leads to qualitatively new results arising solely due to the nonlinearity.

Minor comments:
1) The linear eigenvalue analysis of the EPs on the first page of the SI has been carried in multiple papers, all possible crossings and coalescence of the eigenvalues in such a system are known (e. g. Seyranian et al., 2005 J. Phys. A: Math. Gen. 38 1723).

2) The authors consider a particular example of nonlinearity and show that it maps to a particular catastrophe. In this sense, the results are not universally general. This point is carefully explained in the SM, but we think it should be mentioned in the main text.

Reviewer #2

(Remarks to the Author)
I co-reviewed this manuscript with one of the reviewers who provided the listed reports. This is part of the Nature Communications initiative to facilitate training in peer review and to provide appropriate recognition for Early Career Researchers who co-review manuscripts.

Reviewer #3

(Remarks to the Author)
The authors address the problem of exceptional points (EPs) in nonlinear systems. Their analysis focuses primarily on second-order EPs, while also noting the possibility of extending the results to higher-order cases, particularly in the Supplemental Material (SM). The approach is based on applying results from catastrophe (bifurcation) theory. These standard results are presented in detail in both the main text and the SM, which should facilitate the reader's understanding. The core of the original formulation lies in recasting the eigenvalue problem — central to EP analysis — in terms of an effective potential, with EPs identified as degenerate critical points of this potential. Catastrophe theory is then employed to study how the potential evolves under varying control parameters, beginning, in practice, from what the authors term a linear EP.

Using bifurcation theory and relating bifurcations and the associated dynamical changes to projections of solution surfaces has found multiple applications in physics. This provides the rationale for the paper. However, the presented formulation is confusing.

To begin with, the central term, "nonlinear eigenvalue problem", is used incorrectly, which makes it genuinely difficult to discern what the paper is about. Conventionally, this term refers to the cases where the eigenvalue equation is nonlinear in the eigenvalue. In the present paper it is nonlinear in the eigenvector. This is qualitatively different.

The proposed formulation does not apply to most of the nonlinear dynamical systems typically studied. A possible exception would be systems described by the nonlinear Schrödinger equation with phenomenologically added dissipation and amplification, cf. the paper [58] written by a part of the authors of the present manuscript. However, the inclusion of dissipation necessarily entails noise. In nonlinear systems, noise leads to parameter renormalization and fluctuations, smearing out EPs and strongly affecting higher-order singularities. The paper disregards this major effect.

The paper does not provide an explicit dynamical formulation of the eigenvalue problem. It must instead be inferred by the reader. This further complicates the presentation, in addition to the misuse of the central term. Finally, beyond the physics concerns, the claimed generality of the results is undermined by the use of the potentiality condition. As correctly noted in the SM, the authors assume this condition for their auxiliary function, but it represents a significant limitation for nonlinear dissipative systems.

Given the above concerns, I recommend against publishing the paper in its current form.

Reviewer #4

(Remarks to the Author)
Please see the attached PDF file.

[Editorial Note: See end of file]

Reviewer #5

(Remarks to the Author)
I co-reviewed this manuscript with one of the reviewers who provided the listed reports. This is part of the Nature Communications initiative to facilitate training in peer review and to provide appropriate recognition for Early Career Researchers who co-review manuscripts.

Version 1:

Reviewer comments:

Reviewer #3

(Remarks to the Author)
The paper has been significantly revised and substantially improved. The comments raised in the reports have been addressed. I would have preferred a more detailed discussion of the limitations of the potentiality condition, rather than the current brief statement (“Since, in our case, the Lyapunov potential … exists…”). However, since the authors explicitly acknowledge these limitations while still claiming “universality,” it is probably acceptable to leave the text as it is.

I would also have liked to see more physical background, beyond simply referring to papers [86–89], as the current approach may limit the audience. Nevertheless, I am prepared to leave this decision to the authors.

I would give the authors the opportunity to implement these comparatively minor changes, although the paper could also be recommended for publication in its current form.

(Remarks on code availability)

Reviewer #4

(Remarks to the Author)
Please see attached report.

[Editorial Note: See end of file]

(Remarks on code availability)
The calculations are fairly elementary and, based on the results, seem correct.

**Open Access** This Peer Review File is licensed under a Creative Commons Attribution 4.0 International License, which permits use, sharing, adaptation, distribution and reproduction in any medium or format, as long as you give appropriate credit to the original author(s) and the source, provide a link to the Creative Commons license, and indicate if changes were made.
In cases where reviewers are anonymous, credit should be given to 'Anonymous Referee' and the source.
The images or other third party material in this Peer Review File are included in the article’s Creative Commons license, unless indicated otherwise in a credit line to the material. If material is not included in the article’s Creative Commons license and your intended use is not permitted by statutory regulation or exceeds the permitted use, you will need to obtain permission directly from the copyright holder.
To view a copy of this license, visit https://creativecommons.org/licenses/by/4.0/

**<u>Response to reviewers' reports, manuscript NCOMMS-25-60031-T, Kwong et al., *Universal Topology of Exceptional Points in Nonlinear Non-Hermitian Systems*</u>**

**We would like to thank all 5 reviewers for reviewing our manuscript and their expert opinions. The reviewers have provided insightful reports and constructive criticism. This helped us to make substantial improvements to the manuscript.**

We are grateful that Reviewer #1 says "*the manuscript contains novel and interesting results that justify its publication*", and that "*The manuscript contains interesting novel results beyond this extension. The authors find a catastrophe formed by the exceptional surface within the 3D space of the system's parameters (so each point on its surface corresponds to its own catastrophe), which would not be possible for a linear eigenvalue problem",* and that Reviewer #3 says that "The paper is well written, with extensive supplementary material and references" and remarks that "*The key, interesting aspect here is mapping the eigenvalue equation for the 2 -dimensional complex eigenvector ... into Lyapunov function with no reference to the eigenvalue E*." These summaries indeed capture the main points of our analysis.

While the figures and detailed calculations presented in the paper (both main text and Supplementary Information) have not changed, we were able, in response to the reviewers' remarks, to substantially broaden the claim of the paper. For clarity, the detailed calculations and illustrations of the elliptic umbilic EP structure focus on one example for a nonlinear function (Kerr-type nonlinearity), and in the original manuscript we only mentioned briefly one possible generalization of that example. In the revised version of the manuscript, we broaden the class of matrices with elliptic umbilic nonlinear EP structures to

$$H = \begin{pmatrix} \delta + i\gamma + \alpha F(\mathrm{w}) & b \\ c & -\delta - i\gamma - \alpha F(\mathrm{w}) \end{pmatrix}.$$

with $\delta, \gamma, \in \mathbb{R}, b, c, \alpha \in \mathbb{C}$, and the nonlinearity $F(w)$ being a real-valued analytic function of the eigenvector parameter $w$. Specifically, the real-valued variable $w$ is related to the (right) eigenvector of $H$, which, normalized to 1 , is written as $[xy]^T = (1/A)[1\tilde{w}]^T, \tilde{w} = we^{i\theta}, A = \sqrt{1+w^2}$.

In the original version, the nonlinearity function $F(w)$ was restricted to functions with $F(w) = 0$ and $F'(w) = 0$ at the linear EP. In the new version, we only require that, at the linear EP, $F(w)$ can be approximated by a Taylor expansion. **This class of nonlinearities is clearly much broader than only the Kerr-type nonlinearity that we use in the manuscript to illustrate our findings in terms of a concrete example.**

In fact, **the requirement that the nonlinearity $\boldsymbol{F(w)}$ can be approximated by a Taylor expansion is so general that we would argue that basically all functions that are of practical importance in physics are included in our analysis.** We also clarify that asymmetric matrices ($b \neq c$) are now included in our analysis, i.e. they lead to elliptic umbilic EP structures.

Below, we respond to all comments, questions and concerns in the reviews. The original reports are reproduced in blue font, our response is typed in black font, and red font is used for quotes from our own work.

Accompanying this response is a version of our manuscript where all substantive changes are highlighted in red. The revised manuscript has been formatted according to Nat. Comm. guidance by using the template sn-article.tex.

------------------------------------------------------------------------------------------------------------------------

**Reviewer #1 (Remarks to the Author):**

**We thank reviewer #1 for the insightful review and constructive criticism (possibly written in collaboration with one of the reviewers #2 or #5). The review has prompted us to make changes to the manuscript by (1) adding important references, and (2) substantially broadening the class of nonlinear eigenvalue problems covered by our analysis: the nonlinearity function $F(w)$ can be <u>any</u> function as long as it admits a Taylor expansion at the linear EP.**

**A detailed point-by-point response is given below.**

The authors of the manuscript "Universal Topology of Exceptional Points in Nonlinear Non-Hermitian Systems" study the nonlinear eigenvalue problem for a class of 2x2 non-Hermitian matrices and provide some physical examples that motivate such a study. They analyze exceptional points (EP) that emerge in this setup and link them to the catastrophe theory. In particular, the authors investigate how a manifold of the EPs in the space of system's parameters is deformed due to the nonlinearity compared to the linear eigenvalue problem.

The nonlinear eigenvalue problem that the authors consider consists in finding the eigenvalues and eigenvectors of a non-Hermitian Hamiltonian, which itself explicitly depends on the components of the eigenvectors. This, effectively, reduces to a solution of a system of non-linear equations. The authors analyze a surface of EP locations in the three-dimensional space of system's parameters (a surface of points at which EPs occur). The shape of this surface is connected to a particular type of catastrophes.

For the linear eigenvalue problem, a 2x2 matrix can contain only square root EPs that correspond to the fold catastrophe. In the non-linear regime, the EPs structure can be more involved (since the characteristic equation can be a polynomial of a power higher than 3). For the class of nonlinearities considered by the authors, there exist either two or four exceptional points in different parts of the 3D parametric space, and these regimes are separated by the exceptional surface that corresponds to the elliptic umbilic catastrophe.

The connection between the EPs and the catastrophe theory is quite well-known (as well as the topological consequences of such a connection) for the linear eigenvalue problems. There are many papers on EPs that study this connection implicitly. We mention here a few examples of papers that discuss this connection explicitly: Phys. Rev. B 102, 245144 (2020), Phys. Rev. Lett. 127, 186602 (2021), Nature Phys. 19, 1098–1103 (2023), Phys. Rev. Res. 6, 043209 (2024).

We thank the reviewer for bringing these papers to our attention. These are indeed important works, which are now cited in the revised manuscript. We note that the paper by Delplace et al., Phys. Rev. Lett. 127, 186602 (2021) was already in the original manuscript as Ref. [83]. The paper by Hu et al., Nature Physics 19(8), 1098–1103 (2023)

was already in the original manuscript as Ref. [84]. The papers are still cited, but their reference numbers have changed.

The main novelty claimed by the authors is the extension of the connection between the EPs and the theory of catastrophes to the non-linear case. The manuscript contains interesting novel results beyond this extension. The authors find a catastrophe formed by the exceptional surface within the 3D space of the system's parameters (so each point on its surface corresponds to its own catastrophe), which would not be possible for a linear eigenvalue problem.

This is exactly right. While we have a nonlinear problem in which the number of solutions is in general difficult to determine, we indeed find that only 2 or 4 solutions exist, and the surfaces in parameter space separating the regions of different numbers of solutions is the elliptic umbilic catastrophe.

Regarding the technical details – the analysis is sound and adequate, the calculations presented in the manuscript and the Supplementary Information can be reproduced.
To summarize, the manuscript contains novel and interesting results that justify its publication. The authors generalize a known concept, but this generalization leads to qualitatively new results arising solely due to the nonlinearity.

Minor comments:
1) The linear eigenvalue analysis of the EPs on the first page of the SI has been carried in multiple papers, all possible crossings and coalescence of the eigenvalues in such a system are known (e. g. Seyranian et al., 2005 J. Phys. A: Math. Gen. 38 1723).

This is indeed an important paper that we added to the citations in the first sentence of the introduction.

2) The authors consider a particular example of nonlinearity and show that it maps to a particular catastrophe. In this sense, the results are not universally general. This point is carefully explained in the SM, but we think it should be mentioned in the main text.

The reviewer is right in pointing out that we should state more clearly (in the main text) what functional forms of the nonlinearity are covered by our analysis, in other words, what do we mean when we mention in the abstract "a large class of physical systems". We do this in the revised version of the manuscript in the introduction to Sec. 1 and subsections and 1.2, 1.3, and 1.4.

As mentioned above, we now consider all matrices of the form

$$H = \begin{pmatrix} \delta + i\gamma + \alpha F(\mathrm{w}) & b \\ c & -\delta - i\gamma - \alpha F(\mathrm{w}) \end{pmatrix}.$$

with $\delta, \gamma, \in \mathbb{R}, b, c, \alpha \in \mathbb{C}$, and $F(w)$ being an analytic real-valued nonlinear function of the eigenvector. Specifically, the real-valued variable $w$ is related to the (right) eigenvector of $H$, which, normalized to 1 , is written as $[xy]^T = (1/A)[1\tilde{w}]^T, \tilde{w} = we^{i\theta}, A = \sqrt{1+w^2}$.

As mentioned above, in the original version, the nonlinearity function F(w) was restricted to functions with F(w)=0 and F'(w)=0 at the linear EP. In the new version, we only require that, at the linear EP, F(w) can be approximated by a Taylor expansion. This class of nonlinearities is clearly much broader than only the Kerr-type nonlinearity that we use in the manuscript to illustrate our findings in terms of a concrete example. In fact, the requirement that the nonlinearity F(w) can be approximated by a Taylor expansion is so general that we would argue that basically all functions that are of practical importance in physics are included in our analysis. We also clarify that asymmetric matrices (b≠c) are now included in our analysis, i.e. they lead to elliptic umbilic EP structures.

In Sec. 1.2 of the main text of the revised manuscript, we now discuss all matrices of the form

$$H_1 = \begin{pmatrix} \delta + i\gamma + \alpha_c f(|x|) + \alpha_x f(|y|) & \beta \\ \beta & -\delta - i\gamma - \alpha_x f(|x|) + \alpha_c f|y|) \end{pmatrix}.$$

with real valued nonlinearity functions $f(u)$ that can be, for example, power-law functions, i.e. $f(u) = u^n$ with $n \neq 0$, and where the self-coupling and cross-coupling coefficients $\alpha_c$ and $\alpha_x$, respectively, can be real or imaginary or complex. We also give the example of a periodic function in this class, $f(u) = \cos(u^2)$. Again, **our analysis is not restricted to the Kerr-type nonlinearity of Eq. (1) of the main text; indeed we have infinitely many functional forms included in our analysis, including all power-law functions**.

More details are given in the introduction to Sec. 1 and subsections and 1.2, 1.3 and 1.4 of the revised manuscript.

In the main text of the revised manuscript, we further clarify the terminology, and with it the main claim of our manuscript in Sec 1.2, in the following new paragraph:

Interestingly, we see that the non-trivial topology of the set of EPs in the nonlinear parameters space (the parameter space that includes the nonlinearity $\alpha$, as shown in Fig. 2), is already fully determined by the EPs in the linear space (the subspace of parameters where the nonlinearity is zero). In other words, there are infinitely many possibilities for the EP topology in the nonlinear case (cf. Fig. 1b), but already from the properties of the linear EP alone we find that the topology must be the cone-like structure. At small nonlinearities, this structure is of the form of an elliptic umbilic, i.e. a cone with a deltoid cross section (and the cone-like structure can persist at larger nonlinearities, as in our example, preserving the topology of the elliptic umbilic but not its precise geometry). This is a remarkable statement, since usually one would try to find the set of EPs in the nonlinear parameters space by searching or scanning the part of the parameter space where the nonlinearity is non-zero. To be more specific, if in Eq. (6) we set $\alpha = 0, \beta' = 0$ and $\delta = 0$, we are left with the so-called 'germ' (sometimes called degenerate core) of the catastrophe. For catastrophes with codimension up to 5 (called elementary catastrophes), the germs can be found in textbooks, e.g. [33, 75] and are reproduced in Supplementary Table I. Each germ gives an unambiguous assignment of the corresponding elementary catastrophe. Since, in our case, the Lyapunov potential (as a polynomial in the state variables given by the eigenvectors) that generates the eigenvector equations of the nonlinear eigenvalue problem exists, we can predict the topology of the EP singularity set in the full parameter space solely from the Lyapunov potential where all parameters (including the nonlinearity) have been set to zero. The precise geometric shape can change under smooth transformations of parameters, but its topological

properties are determined by the universal unfolding (which is defined as the versal unfolding of minimal dimension, see, e.g., p. 621 of Ref. [75]) of the elliptic umbilic singularity.

Finally, we provide two tables meant to illustrate the generality (universality) of our results which, in the revised manuscript, is discussed in much greater detail and - we believe - made much clearer:

| Categories now explicitly mentioned | Original manuscript | Revised manuscript |
|---|---|---|
| $F(1) = 0$ and $F'(1) = 0$ | ✗ | ✓ |
| $F(1) = 0$ and $F'(1) \neq 0$ | ✓ | ✓ |
| $F(1) \neq 0$ and $F'(1) = 0$ | ✗ | ✓ |
| $F(1) \neq 0$ and $F'(1) \neq 0$ | ✗ | ✓ |
| Here, the nonlinearity in H is assumed to be written as $\alpha F(w)$ and $F'(w) = dF/dw$ | | |

| Examples within category $F$(1)=0 and $F'$(1)≠0 | Original manuscript | Revised manuscript |
|---|---|---|
| quadratic nonlinearity $f(u) = u^2$ | ✓ | ✓ |
| power law nonlinearity $f(u) = u^n$ $(n \neq 0)$<br>oscillatory nonlinearity $f(u) = cos(u^2)$ | ✗ | ✓ |
| nonlinear coefficient $\alpha$ real | ✓ | ✓ |
| nonlinear coefficient $\alpha$ complex | ✗ | ✓ |
| symmetric matrix, off-diagonal $b = c$ real | ✓ | ✓ |
| symmetric matrix, off-diagonal $b = c$ complex | ✗ | ✓ |
| asymmetric matrix, off-diagonal $b, c$ arbitrary complex | ✗ | ✓ |
| Here, the nonlinearity in H is assumed to be written as $\alpha F(w) = \alpha(f(\lvert x\rvert) - f(\lvert y\rvert))$<br>The off-diagonal elements are written as $\begin{pmatrix} \cdots & b \\ c & \cdots \end{pmatrix}$ | | |

We hope that we have sufficiently addressed all the Reviewer's concerns.

**Reviewer #2 (Remarks to the Author):**

**We thank the reviewer for reviewing our manuscript.**

I co-reviewed this manuscript with one of the reviewers who provided the listed reports. This is part of the Nature Communications initiative to facilitate training in peer review and to provide appropriate recognition for Early Career Researchers who co-review manuscripts.

**Reviewer #3 (Remarks to the Author):**

**We thank reviewer #3 for the insightful review and constructive criticism (possibly written in collaboration with one of reviewers #2 or #5). The review has prompted us to make changes to the main text addressing the issues of (1) clarification in what sense we use the term 'nonlinear eigenvalue problem', (2) adding important references, (3) adding clarifications regarding noise and dynamical evolution equations, and (4) substantially broadening the class of nonlinear eigenvalue problems covered by our analysis: the nonlinearity function $F(w)$ can be <u>any</u> function as long as it admits a Taylor expansion at the linear EP.**

**A detailed point-by-point response is given below.**

The authors address the problem of exceptional points (EPs) in nonlinear systems. Their analysis focuses primarily on second-order EPs, while also noting the possibility of extending the results to higher-order cases, particularly in the Supplemental Material (SM). The approach is based on applying results from catastrophe (bifurcation) theory. These standard results are presented in detail in both the main text and the SM, which should facilitate the reader's understanding. The core of the original formulation lies in recasting the eigenvalue problem — central to EP analysis — in terms of an effective potential, with EPs identified as degenerate critical points of this potential. Catastrophe theory is then employed to study how the potential evolves under varying control parameters, beginning, in practice, from what the authors term a linear EP.

Using bifurcation theory and relating bifurcations and the associated dynamical changes to projections of solution surfaces has found multiple applications in physics. This provides the rationale for the paper. However, the presented formulation is confusing.

To begin with, the central term, "nonlinear eigenvalue problem", is used incorrectly, which makes it genuinely difficult to discern what the paper is about. Conventionally, this term refers to the cases where the eigenvalue equation is nonlinear in the eigenvalue. In the present paper it is nonlinear in the eigenvector. This is qualitatively different.

We are grateful to the reviewer for noting a potential misunderstanding. In the literature, the term '*nonlinear eigenvalue problem*' is used in various cases or contexts. Among the most common cases are the one mentioned by the reviewer (namely a matrix eigenvalue problem where the matrix depends on the eigenvalue) and the case where the matrix depends on the eigenvector. It is also possible that the matrix depends on both, the eigenvalue and the eigenvectors. In some instances or approximations, analytical expressions for the nonlinearity can be given in terms of the eigenvector and then equivalently the eigenvalue, as for example in the paper by Zheng and Chong, *Noise Constraints for Nonlinear Exceptional Point Sensing*, Phys. Rev. Lett. 134, 133801 (2025),

https://journals.aps.org/prl/abstract/10.1103/PhysRevLett.134.133801

Nonlinear eigenvalue problems in a formally different context have been analyzed in detail by Carl Bender (the pioneer of research on PT-symmetry), see for example

Bender C M, Fring A, and Komijani J (2014) *Nonlinear eigenvalue problems* J. Phys. A: Math. Theor. 47 235204

https://iopscience.iop.org/article/10.1088/1751-8113/47/23/235204

and

Carl M. Bender, *Nonlinear eigenvalue problems and PT -symmetric quantum mechanics*, IOP Conf. Series: Journal of Physics: Conf. Series 873 (2017) 012002

https://iopscience.iop.org/article/10.1088/1742-6596/873/1/012002

In light of the various definitions of the term '*nonlinear eigenvalue problem*' we agree with the reviewer that it is beneficial to clearly state which case we are dealing with.

For example, in a recent paper by Isobe et al., *Bulk-Edge Correspondence for Nonlinear Eigenvalue Problems*, Phys. Rev. Lett. 132, 126601 (2024),

https://journals.aps.org/prl/abstract/10.1103/PhysRevLett.132.126601

the nonlinear matrix does indeed depend on the eigenvalue, as the reviewer points out. There may be other important publications in this category that we should cite (but are presently unaware of) when delineating the eigenvalue problem used in our manuscript and contrasting it with those mentioned by the reviewer. In our revised manuscript, we follow the example of Isobe et al., who are aware of the possible misunderstanding, and who therefore note that "*In Ref. [83], the word "nonlinear eigenvalue problem" is employed to describe the nonlinearity of eigenvectors, which differs from the terminology in this Letter*" where Ref. [83] is the paper by Sone et al., *Topological synchronization of coupled nonlinear oscillators,* Phys. Rev. Research 4, 023211, 2022

https://journals.aps.org/prresearch/abstract/10.1103/PhysRevResearch.4.023211

The Sone et al. paper is a rather general study of "topological nonlinear oscillators", and the matrix governing the system's dynamics through a set of ordinary first-order time-differential equations has a quadratic nonlinearity similar to the Kerr-like nonlinearity that we use in our paper to illustrate the topological properties of the EP geometric object in parameter space (which we find to be a quasi-deltoid cone-like non-differentiable manifold).

We have included an abbreviated version of this discussion together with the references in the revised version of the manuscript:

The underlying eigenvalue problem here may be nonlinear in the sense that the matrix $M$ may depend on the eigenvalue $\lambda$ (e.g. [68]), the eigenvector x (e.g. [69]), or both. In some instances, the nonlinear dependence on the eigenvector can be interchanged by that on the eigenvalue, e.g. [34]. Other kinds of nonlinear eigenvalue problems are discussed in [70]. In the following, we concentrate on the case where the nonlinear eigenvalue problem is formulated as a matrix equation where the matrix dependends on the eigenvector. For example, the energies in an optical microcavity, which are described by the diagonal elements of $M$, can shift with the intensity of the light field described by $\mathbf{x}$, hence $M(\mathbf{x})\mathbf{x} = \lambda\mathbf{x}$. In the following, we refer to EPs that are solutions of a linear eigenvalue problem as 'linear EPs,' and those solving a nonlinear eigenvalue problem are called 'nonlinear EPs.'

The proposed formulation does not apply to most of the nonlinear dynamical systems typically studied. A possible exception would be systems described by the nonlinear Schrödinger equation with phenomenologically added dissipation and amplification, cf. the paper [58] written by a part of the authors of the present manuscript. However, the inclusion of dissipation necessarily entails noise. In nonlinear systems, noise leads to parameter renormalization and fluctuations, smearing out EPs and strongly affecting higher-order singularities. The paper disregards this major effect.

Our work does apply to a large class of nonlinear dynamical systems, but we agree with the reviewer that we did not clearly identify what this means in mathematical terms. We do this in the revised version of the manuscript in the introduction to Sec. 1 and the subsections Sec. 1.2, 1.3 and 1.4.

As mentioned above, we clearly say now that our work covers all matrices of the form

$$H = \begin{pmatrix} \delta + i\gamma + \alpha F(\mathrm{w}) & b \\ c & -\delta - i\gamma - \alpha F(\mathrm{w}) \end{pmatrix}.$$

with $\delta, \gamma, \in \mathbb{R}, b, c, \alpha \in \mathbb{C}$, and the nonlinearity $F(w)$ being a real-valued analytic function of the eigenvector parameter $w$. Specifically, the real-valued variable $w$ is related to the (right) eigenvector of $H$, which, normalized to 1 , is written as $[xy]^T = (1/A)[1\tilde{w}]^T, \tilde{w} = we^{i\theta}, A = \sqrt{1+w^2}$.

Furthermore, as mentioned above, in the original version, the nonlinearity function $F(w)$ was restricted to functions with $F(w) = 0$ and $F'(w) = 0$ at the linear EP. In the new version, we only require that, at the linear EP, $F(w)$ can be approximated by a Taylor expansion. **This class of nonlinearities is clearly much broader than only the Kerr-type nonlinearity that we use in the manuscript to illustrate our findings in terms of a concrete example.** In fact, **the requirement that the nonlinearity $F(w)$ can be approximated by a Taylor expansion is so general that we would argue that basically all functions that are of practical importance in physics are included in our analysis.** We also clarify that asymmetric matrices ($b \neq c$) are now included in our analysis, i.e. they lead to elliptic umbilic EP structures.

In Sec. 1.2 of the main text of the revised manuscript, we now discuss in detail matrices of the form

$$H_1 = \begin{pmatrix} \delta + i\gamma + \alpha_c f(|x|) + \alpha_x f(|y|) & \beta \\ \beta & -\delta - i\gamma - \alpha_x f(|x|) + \alpha_c f|y|) \end{pmatrix}.$$

with real valued nonlinearity functions $f(u)$ that can be, for example, power-law function, i.e. $f(u) = u^n$ with $n \neq 0$, and where the self-coupling and cross-coupling coefficients $\alpha_c$ and $\alpha_x$, respectively, can be real or imaginary or complex. We also give the example of a periodic function in this class, $f(u) = \cos(u^2)$. In other words, **our analysis is not restricted to the Kerr-type nonlinearity of Eq. (1) of the main text, but includes infinitely many functional forms, including all power-law functions**.

More details are given in Sec. 1.2 of the revised manuscript.

The reviewer correctly points out that noise is an important aspect of studies of EPs. In particular in the context of quantum sensing, which we had explicitly mentioned in our manuscript, there is an ongoing scientific debate over the precise role noise plays in the enhancement of measurable quantities in the vicinity of an EP. It is then also important to understand the interplay between noise and nonlinearity if the system's response is nonlinear.

One recent paper that addresses the renormalization or displacement of an EP in parameter space due to noise is the above-mentioned paper by Zheng and Chong, *Noise Constraints for Nonlinear Exceptional Point Sensing*, Phys. Rev. Lett. 134, 133801 (2025),

https://journals.aps.org/prl/abstract/10.1103/PhysRevLett.134.133801

It is shown in that paper that, if the noise-free solution of the nonlinear system is known (in their case the 2x2 matrix $H$ in Eq. (55) and (S21) of their paper), then the interplay of noise and nonlinearity can be treated by a 4x4 matrix $H_B$, Eq. (19) of their paper.

However, the problem here is that **we first need to obtain the solutions of the nonlinear equations without noise**. Zheng and Chong find solutions only for the specific approximation of real-valued eigenvalues. On the other hand, our analysis covers their model and therefore we do indeed present the full nonlinear solution without noise. We are not aware of any publications that provide the full nonlinear solutions. **It is then clear that only our solutions can provide the basis for a future general treatment of the interplay between noise and nonlinearity.**

Another interesting study of the interplay between noise and nonlinearity was given by Suntharalingam et al. in a recent Nature Communication paper, *Noise resilient exceptional-point voltmeters enabled by oscillation quenching phenomena*, Nature Communications ( 2023) 14:5515,

https://www.nature.com/articles/s41467-023-41189-7

as well as the papers by Bai et al. which are Refs. [62.63] in the original version of our manuscript.

The role of noise has also been studied in 3rd-order EPs, for example in the paper by Panahi et al. titled *Higher-order exceptional points and stochastic resonance in pseudo-Hermitian systems,* Phys. Rev. Appl. 22, 054063 (2024).

https://journals.aps.org/prapplied/abstract/10.1103/PhysRevApplied.22.054063

The papers by Zheng and Chong , Suntharalingam et al. and Panahi et al. are now cited in the revised version of the manuscript.

To summarize and clarify our response to this question, **our work provides an important and general tool for the analysis of the interplay between noise and nonlinearity** in the following sense. Let us assume we have a system that can be modeled by a nonlinear eigenvalue problem, and we want to understand the influence of noise. Then we must first find the EPs in parameter space without noise, and then, for a given EP, we can add the noise as a perturbation. This leads us to the question: where in the nonlinear parameter space are the EPs, and what characteristics do they have, for example: do they behave like 2nd or 3rd order nonlinear EPs; are other EPs in their immediate neighborhood; what is the dimension of the neighborhood; if there are adjacent EPs, do they form a line, a surface, a volume, a hyper-surface; can the neighborhood be classified as a differentiable manifold or surface, etc.? If we are not able to answer these questions, we cannot obtain a comprehensive understanding of the role of noise. And this is exactly what our paper provides, because we can answer all of these questions for a large (infinite) class of nonlinearities for which we can find a Lyapunov potential that generates the eigenvalue equations.

In other words, we are not disregarding effects related to noise, we are enabling studying those effects for a large (infinite) class of nonlinearities. In Sec 2 (Discussion) of the revised version of the manuscript we now say

Our prediction of the singularity structure of EPs in the nonlinear regime should enable systematic studies and possible optimization of the renormalization of EPs due to noise and stochastic processes, e.g. [34, 57, 66, 92].

The paper does not provide an explicit dynamical formulation of the eigenvalue problem. It must instead be inferred by the reader. This further complicates the presentation, in addition to the misuse of the central term. Finally, beyond the physics concerns, the claimed generality of the results is undermined by the use of the potentiality condition. As correctly noted in the SM, the authors assume this condition for their auxiliary function, but it represents a significant limitation for nonlinear dissipative systems.

In the original version of the manuscript we just gave one example of a possible dynamic equation for which our analysis is useful. There we said:

Examples of systems that have been analyzed by catastrophe theory include dynamical systems obeying equations of motion of the form $\dot{\mathbf{x}} = -\mathrm{grad}\{V_L(\mathbf{x})\}$,

and further down in that paragraph we said

Similarly, in optics, the solution of the Helmholtz equation....

However, we agree with the reviewer that some additional discussion could further clarify this point. In the revised version of the manuscript, we now also say

...the solution of the Helmholtz equation, which described the stationary solutions of a second-order time-differential equation, in contrast to the example of a first-order time-differential equation mentioned above, can be evaluated ...

Regarding the issue of generality, we agree that this needed further clarification. As mentioned above, in the introduction to this response letter and in response also to Reviewers #1 and #4, we have included in the introduction to Sec. 1 and in subsections 1.2, 1.3 and 1.4 of the revised manuscript a detailed discussion of the class of matrices covered by our analysis, and stress that **our analysis is not restricted to the Kerr-type nonlinearity of Eq. (1) of the main text in the original manuscript.** As mentioned above, in the revised manuscript, **the requirement that the nonlinearity *F(w)* can be approximated by a Taylor expansion is so general that we would argue that basically all functions that are of practical importance in physics are included in our analysis.**

We further clarify the terminology, and more importantly main claim of our manuscript, in the following new paragraph:

Interestingly, we see that the non-trivial topology of the set of EPs in the nonlinear parameters space (the parameter space that includes the nonlinearity $\alpha$, as shown in Fig. 2), is already fully determined by the EPs in the linear space (the subspace of parameters where the nonlinearity is zero). In other words, there are infinitely many possibilities for the EP topology in the nonlinear case (cf. Fig. 1b), but already from the properties of the linear EP alone we find that the topology must be the cone-like structure. At small nonlinearities, this structure is of the form of an elliptic umbilic, i.e. a cone with a deltoid cross section (and the cone-like structure can persist at larger nonlinearities, as in our example, preserving the topology of the elliptic umbilic but not its precise geometry). This is a remarkable statement, since usually one would try to find the set of EPs in the nonlinear parameters space by searching or scanning the part of the parameter space where the nonlinearity is non-zero. To be more specific, if in Eq. (6) we set $\alpha = 0, \beta' = 0$ and $\delta = 0$, we are left with the so-called 'germ' (sometimes called degenerate core) of the catastrophe. For catastrophes with codimension up to 5 (called elementary catastrophes), the germs can be found in textbooks, e.g. [33, 75] and are reproduced in Supplementary Table I. Each germ gives an unambiguous assignment of the corresponding elementary catastrophe. Since, in our case, the Lyapunov potential (as a polynomial in the state variables given by the eigenvectors) that generates the eigenvector equations of the nonlinear eigenvalue problem exists, we can predict the topology of the EP singularity set in the full parameter space solely from the Lyapunov potential where all parameters (including the nonlinearity) have been set to zero. The precise geometric shape can change under smooth transformations of parameters, but its topological properties are determined by the universal unfolding (which is defined as the versal unfolding of minimal dimension, see, e.g., p. 621 of Ref. [75]) of the elliptic umbilic singularity.

Regarding the use of the term 'universal' in the title, delineating the meaning of a technical term used in the title (here 'universal') through explanations in the abstract and the main text is in line with common practice in Nat. Comm. and other high-profile journals.

For example, the recent paper by Kilic et al., '***Universal** symmetry-protected persistent spin textures in noncentrosymmetric crystals*', Nat. Comm. (2025) 16:7999 (2025), clarifies in the abstract that the word 'universal' in the title is to be understood **with the exception** of all crystals in the space group P1, even though they are noncentrosymmetric. Another example is '***Universality** of Dicke superradiance in arrays of quantum emitters*' by Masson and Asenjo-Garcia, Nat. Comm. 13:2285 (2022), where the analysis is **restricted** to Markovian system, leaving out all systems with quantum memory effects.

Finally, we provide two tables meant to illustrate the generality (universality) of our results which, in the revised manuscript, is discussed in much greater detail and - we believe - made much clearer:

| Categories now explicitly mentioned | Original manuscript | Revised manuscript |
|---|---|---|
| $F(1) = 0$ and $F'(1) = 0$ | ✗ | ✓ |
| $F(1) = 0$ and $F'(1) \neq 0$ | ✓ | ✓ |
| $F(1) \neq 0$ and $F'(1) = 0$ | ✗ | ✓ |
| $F(1) \neq 0$ and $F'(1) \neq 0$ | ✗ | ✓ |
| Here, the nonlinearity in H is assumed to be written as $\alpha F(w)$ and $F'(w) = dF/dw$ | | |

| Examples within category $F(1)$=0 and $F'(1)$≠0 | Original manuscript | Revised manuscript |
|---|---|---|
| quadratic nonlinearity $f(u) = u^2$ | ✓ | ✓ |
| power law nonlinearity $f(u) = u^n$ $(n \neq 0)$<br>oscillatory nonlinearity $f(u) = cos(u^2)$ | ✗ | ✓ |
| nonlinear coefficient $\alpha$ real | ✓ | ✓ |
| nonlinear coefficient $\alpha$ complex | ✗ | ✓ |
| symmetric matrix, off-diagonal $b = c$ real | ✓ | ✓ |
| symmetric matrix, off-diagonal $b = c$ complex | ✗ | ✓ |
| asymmetric matrix, off-diagonal $b, c$ arbitrary complex | ✗ | ✓ |
| Here, the nonlinearity in H is assumed to be written as $\alpha F(w) = \alpha(f(\lvert x \rvert) - f(\lvert y \rvert))$<br>The off-diagonal elements are written as $\begin{pmatrix} \cdots & b \\ c & \cdots \end{pmatrix}$ | | |

Given the above concerns, I recommend against publishing the paper in its current form.

We hope that the clarifications we give in response to all of the Reviewer's concerns will suffice for the Reviewer to revise their recommendation.

**Reviewer #4 (Remarks to the Author):**

EDITOR'S EMAILS: Please see the attached PDF file.
AUTHOR'R REMARK: For the purposes of this response, we have transcribed the pdf file containing the review to MS Word format.

**We thank reviewer #4 for the insightful review and constructive criticism (possibly written in collaboration with one of reviewers #2 or #5). The review has prompted us to make changes to the main text addressing the issues of (1) eigenvector coalescence, (2) parallels to the nonlinear Schrödinger equation and new insight on**

**symmetries regarding nonlinear parameters, (3) clarifications regarding topological properties vs. uniqueness of the solutions, (4) validity for higher order nonlinearities (including quartic) and for coupling parameters that are real (nonlinear phase shift) and imaginary (nonlinear absorption), and (5) clarification of the term "nonlinear topology."**

**A detailed point-by-point response is given below.**

Report on "Universal topology of exceptional points in nonlinear non-Hermitian systems" by N.H. Kwong et al.

In this article, the authors consider a two-state dynamical system described by a non-Hermitian generator of time evolution H . The eigenvalue problem for such nonlinear H is recast in terms of Lyapunov function, whose vanishing gradient generates the eigenvalue equations and whose vanishing Hessian gives the exceptional point degeneracies of the nonlinear Hamiltonian. By using Kerr-type (cubic) nonlinearity, the Lyapunov function is constructed and shown to match the elliptic-umbilic category from catastrophe theory. The authors obtain analytical and numerical results for the evolution of nonlinear EPs as a function of the nonlinearity strength. Then the authors claim that the results are generalizable to other nonlinearities.

The paper is well written, with extensive supplementary material and references. The key, interesting aspect here is mapping the eigenvalue equation for the 2 -dimensional complex eigenvector, characterized by ( $w, \theta$ ), into Lyapunov function with no reference to the eigenvalue $E$, so that the results in the classical Catastrophe theory with two variables (and number of parameters, three in this case, corresponding to dimensionless coupling $\beta / \gamma$, dimensionless detuning $\delta / \gamma$, and dimensionless nonlinearity $\alpha / \gamma$ ). This mapping onto specific, elliptic umbilic topology is clear for the Kerr-nonlinearity problem they have considered; however, we have several concerns regarding the claim of "universal topology". The following need to be addressed before the paper can be further considered:

1. The discussion is focused on eigenvalue degeneracy criterion. When claiming EPs, as understood in the non-Hermitian community, the authors should also show corresponding eigenvector coalescence. In linear models, it is easy to create examples where a 3 -fold eigenvalue degeneracy has two independent eigenvectors, thereby having only EP2.

   To clarify this, we would like to note that, in the reviewer's next item (2), the reviewer correctly summarizes our approach: "*The authors create a Lyapunov potential that generates the eigenvalue equation* ". What is meant by "*generates*" is that the equations for the singularity set (i.e. set of degenerate critical points) of the potential, where both the gradient and the Hessian are zero, are the same as the eigenvector equations (where by 'eigenvector equations' we mean the equations for zero determinant with the eigenvalue eliminated). Hence, by design, the eigenvectors coalesce when the critical points are degenerate. These equations are equations for the state variables (in our case the eigenvector norm related to the parameter that we call *w* and phase $\theta$) and the control parameters. The EP object (the deltoid cone) that we show in Fig. 2 (sometimes called bifurcation set) is then obtained by eliminating the state variables (in other words, by projecting the 'hyper-surface' defined by the eigenvector equations into the control parameter space). Every point on that cone-like bifurcation set has at least a second order EP, so again, by design, the eigenvectors do coalesce at those parameter values. Of course, since in our case the Hilbert space is 2-dimensional, we can at most have 2nd-order EPs in the linear case. But in the nonlinear case, we find that we can either have 2 or 4 (but not other) solutions to the eigenvector equations, so we have 2nd, 3rd and 4th order *nonlinear EPs (with the 3rd order nonlinear EPs* on the cusps of the cone and the 4th-order nonlinear EP in the limit of vanishing nonlinearity). Note

that, in the case of 4 solutions, we have 4 eigenvectors coalesce, but they are linearly dependent, since our Hilbert space is only 2-dimensional. This is very different from the linear case, where, as the reviewer correctly points out, the degree of degeneracy is at most the dimension of the Hilbert space which, in general, is equal to or greater than the order of the EP. Indeed, some of us have recently shown that a 3x3 matrix for the fluctuation modes in a semiconductor laser can have fold lines of EP2s that merge at a cusp point that is an EP3 (Kwong et al., PRB 109, 045306 (2024) Editors' Suggestion). The physics underlying this is a gapless-to-gapped transition in the fluctuation mode spectrum. A more extreme case where the Hilbert space is much larger than the order of the EP is where we use a continuum model (in the numerics the matrix is typically 1000x1000) and we were able to find an EP2 from this matrix, which is not trivial, given the large difference between the Hilbert space dimension and the order of the EP (Binder, Kwong, PRB 103, 085304 (2021)).

In the revised version of the manuscript, we now say:

We also note that in higher dimensional systems (described by $N \times N$ matrices with $N > 2$ ), even in the linear case we can have EPs of various orders, for example a $3 \times 3$ matrix can have second and third-order linear EPs. The structure of the nonlinear EP can then be expected to be more complex than the case of nonlinear EPs discussed above for the $2 \times 2$ matrix. We found that in the $2 \times 2$ case where a Lyapunov potential exists, the coalescence of eigenvectors agrees with the condition of singularities of the Lyapunov function, and - as is already known [65-67] - the order of the nonlinear EPs can be higher than that of the linear EPs (in our case, the linear problem can only have second order EPs while the nonlinear problem has 4th-order nonlinear EPs).

2. The authors create a Lyapunov potential that generates the eigenvalue equation. However, the uniqueness of this potential is not addressed. Specifically, different potentials can give rise to different Hessians and thereby potentially different EP landscapes. The authors should address this in the main text. In dissipative systems, many different Lagrangians can give rise to the same equations of motion (for example, mass + spring system with time-dependent mass vs. Bateman oscillators, both give rise to the equations of motion for a damped harmonic oscillator.)

The reviewer is right in pointing out that different Hessians are generally creating different EP landscapes. And in our paper, we show only one such landscape, namely the cone-like structure in Fig. 2. And the fact that we show only one landscape is probably the strongest and most interesting aspect of our work. We are claiming that all solutions covered by our analysis (and defined in the introduction to Sec. 1 and subsections 1.2, 1.3 and 1.4 of the revised manuscript) lead to EP objects that are topologically equivalent to the EP object shown in Fig. 2. This is a very strong statement. It says that for any nonlinearity $F(w)$ one can generate a bifurcation set (and there are infinitely many such sets covered by our analysis), but for all of those different bifurcation sets there exists a smooth parameter transformation that transforms those different bifurcation sets into the deltoid cone shown in Fig. 2. We want to stress that this statement is much stronger than usual statements obtained from bifurcation theory, and it is only valid because we have shown that the Lyapunov function exists, and therefore Thom's theorem can be applied. Roughly speaking, Thom's theorem tells us that if we have up to five control parameters in the Lyapunov potential, then we know that the bifurcation set (or the 'catastrophe') must be one of the elementary catastrophes listed in Tab. 1 of the Supplementary Information. All we have to do is to find the 'germ' in order to unambiguously identify the elementary catastrophe. Finding the germ is, in practice, very difficult, just as finding the Lyapunov potential. This is because the Lyapunov potential may need a nontrivial smooth

parameter transformation before one can see and identify the germ. But once we are able to find the Lyapunov potential and identify the germ, we can identify the entire bifurcation set. And this is exactly what we have done in this work.

We hope that this explanation helps the reviewer to appreciate the substantial scientific progress that our manuscript provides.

In the revised manuscript, we say (the following is an excerpt from a paragraph that we have given in its entirety in our response to one of the later comments by this Reviewer (#4)):

Since, in our case, the Lyapunov potential (as a polynomial in the state variables given by the eigenvectors) that generates the eigenvector equations of the nonlinear eigenvalue problem exists, we can predict the topology of the EP singularity set in the full parameter space solely from the Lyapunov potential where all parameters (including the nonlinearity) have been set to zero. The precise geometric shape can change under smooth transformations of parameters, but its topological properties are determined by the universal unfolding (which is defined as the minimal versal unfolding) of the elliptic umbilic singularity.

We also note that the interesting case of the Bateman oscillators mentioned by the reviewer is related to PT symmetry, which we indicate by the thick blue line in Fig. 3 of the main text.

3. The paper discussion is rigorous for cubic nonlinearities in the Hermitian part of the Hamiltonian that arise from the Kerr effect. However, they do not encompass major physically relevant or mathematically interesting cases. Notable absences include the following. a) The gain-saturation nonlinearity that shows up in the anti-Hermitian part of the Hamiltonian, and is asymmetric meaning only arises for the gain side, not the loss side. b) In the Hermitian part, higher-order nonlinearities - particularly a quartic one - are relevant; see, for example, nonlinear optics by Robert Boyd chapter 1. c) In a system like a nonlinear pendulum, or its quantum equivalent in a superconducting circuit, the nonlinearity is a periodic function of the dynamical variable.

We will try our best to address this item, but with the limited information given we may fall short of satisfying the Reviewer's expectations. We assume the reviewer refers to Eq. (1) of the main text with the matrix reproduced by Eq. (10) of the original Supplementary Information, with real $\beta$ . Then, we believe that the reviewer refers to the term $\pm\alpha(|x|^2 - |y|^2)$ as gain saturation nonlinearity. In the main text, $\alpha$ is taken to be real-valued, hence it is related to a nonlinear refractive index (in the language of classical optical systems) or nonlinear energy shifts. However, in the original Supplementary Information we had also analyzed the case where $\alpha$ is imaginary, which could be called gain saturation and is probably what the reviewer has in mind. This case is indeed included in our class of matrices and therefore has an elliptic umbilic catastrophe. This was discussed in the original Supplementary Information after Eq. (15), where we said "A similar analysis for the case that α is purely imaginary yields a EU germ similar to the one in the case of α being purely real, but with ϕ and r interchanged. In this case, the catastrophe remains elliptic umbilic." Unfortunately, we do not understand what the reviewer means by "asymmetric meaning only arises for the gain side, not the loss side", we would need to have that statement clarified in terms of mathematical formulas in order to give a satisfactory response.

Regarding (b) and (c), based on the reviewer's suggestion, we have extended our analysis and now discuss a broad class of nonlinearity functions $f(u)$ entering the matrix. In the original manuscript, it was not sufficiently clear in what 'large class' (in the abstract) really meant, and we did not provide a mathematically well-defined class of nonlinearity functions that would satisfy the condition $F(1) = 0$ and $F'(1) \neq 0$.

As mentioned above, our work now covers all matrices of the form

$$H = \begin{pmatrix} \delta + i\gamma + \alpha F(\mathrm{w}) & b \\ c & -\delta - i\gamma - \alpha F(\mathrm{w}) \end{pmatrix}.$$

with $\delta, \gamma, \in \mathbb{R}, b, c, \alpha \in \mathbb{C}$, and the nonlinearity $F(w)$ being a real-valued analytic function of the eigenvector parameter $w$. Specifically, the real-valued variable $w$ is related to the (right) eigenvector of $H$, which, normalized to 1 , is written as $[xy]^T = (1/A)[1\tilde{w}]^T, \tilde{w} = we^{i\theta}, A = \sqrt{1+w^2}$.

Furthermore, as mentioned above, in the original version, the nonlinearity function $F(w)$ was restricted to functions with $F(w) = 0$ and $F'(w) = 0$ at the linear EP. In the new version, we only require that, at the linear EP, $F(w)$ can be approximated by a Taylor expansion. **This class of nonlinearities is clearly much broader than only the Kerr-type nonlinearity that we use in the manuscript to illustrate our findings in terms of a concrete example.** In fact, **the requirement that the nonlinearity $F(w)$ can be approximated by a Taylor expansion is so general that we would argue that basically all functions that are of practical importance in physics are included in our analysis.** We also clarify that asymmetric matrices ($b \neq c$) are now included in our analysis, i.e. they lead to elliptic umbilic EP structures.

Furthermore, in the revised manuscript, Sec. 1.2 *Extension beyond quadratic nonlinearities*, we now discuss matrices of the form

$$H_1 = \begin{pmatrix} \delta + i\gamma + \alpha_c f(|x|) + \alpha_x f(|y|) & \beta \\ \beta & -\delta - i\gamma - \alpha_x f(|x|) + \alpha_c f|y|) \end{pmatrix}.$$

with real valued nonlinearity functions $f(u)$ that a power-law function, i.e. $f(u) = \mathrm{u}^{\mathrm{n}}$ with $n \neq 0$, and where the self-coupling and cross-coupling coefficients $\alpha_c$ and $\alpha_x$, respectively, can be real or imaginary or complex. We also give the example of a periodic function in this class, $f(u) = \cos(u^2)$. We note, however, that our analysis, similar to many other bifurcation studies, is focusing on neighborhoods of EPs or more generally bifurcations. In the case of a nonlinearity that is periodic in a certain parameter (in our case the state variables provided by the eigenvector elements), then one would probably perform a Taylor expansion around the EP and again have a power dependence rather than a periodic dependence. We also note that the physics of many nonlinear oscillators is well established and textbook discussion regarding the applicability of Catastrophe theory are available. This included the Duffing oscillator and the van der Pol oscillator, which can only be approximately related to a cusp catastrophe, see p. 70 of the book by Saunders, Ref. [67] of the original manuscript.

To summarize this point, we want to stress again that **our analysis is not restricted to the Kerr-type nonlinearity of Eq. (1) of the main text, but includes infinitely many functional forms, including all power-law functions**.

More details are given in the introduction to Sec. 1 and subsections and 1.2, 1.3 and 1.4 of the revised manuscript.

4. In the current manuscript, the results provided are symmetric in sign of nonlinearity, $\alpha/\gamma$. However, in the Kerr model for the continuum nonlinear Schrodinger equation, positive and negative signs refer to physically different effects (focusing vs. non-focusing). The authors should comment on the seeming presence of this symmetry.

   This is an interesting question, which requires knowledge of whether the "Kerr model for the continuum nonlinear Schrodinger equation" can be mapped to a 2x2 nonlinear matrix equation, and what exactly the dynamic solutions of the nonlinear 2x2 model are. We do think that posing this question in the main text improves our manuscript, and therefore we included the following discussion, in which we reiterate the symmetry properties of the EP structure and clarify that the symmetry is with respect to the difference between the self and cross coupling coefficients. If the self and cross couplings are equal, the nonlinearity contributes only a multiple of the unit matrix and therefore does not lead to the elliptic umbilic structure shown in Fig. 2.

   Here is the new discussion, included in the manuscript as Sec. 1.5:

   We also mention again that the EP structure shown in Fig. 2 is symmetric with respect to the Kerr-like nonlinearity $\alpha$ and the linear coupling $\beta$. For real-valued self coupling ($\alpha_c$) and cross coupling ($\alpha_x$) in Eq. (6), it is instructive to compare our nonlinear $2 \times 2$ eigenvalue problem with the nonlinear Schrödinger equation (NLS). If the NLS is derived from the Helmholtz equation in paraxial approximation, then the spatial propagation coordinate $z$ plays the role of time and the diffraction (second-order derivative in the transverse direction) plays the role of the kinetic energy, see e.g. [90]. While the general solution structure of the NLS includes effects like soliton formation and is generally complicated (and the NSL is not integrable in more than one spatial dimensions), it has been shown in [91] that coupled-mode analysis in the nonlinear regime can be done with the NLS under suitable conditions. If one would use two modes with different transverse extensions, effects analogous to self-focusing and self-defocusing could be described by a 2 -mode model. In the language of our eigenvalue equation, the eigenvalue $E$ would correspond to the wave vector in the propagation direction $z$, and the coupling coefficients $\alpha_c$ and $\alpha_x$ would depend on the spatial overlap between the two modes. More detailed studies of the discrete NLS (also called discrete self-trapping equation) are provided in Ref. [92], but most studies of the discrete NLS, including 2 -mode models that are equivalent to the (nonlinear) pendulum [93], do not include decay, which is essential in our study of the EP structure. If, however, a self-focusing and self-defocusing system with decay can be described by our 2-mode model, Eq. (6), with the difference in the absorption coefficients of the two modes given by $\gamma$, then we predict that the EP structure is symmetric in $\alpha = \frac{1}{2}(\alpha_c - \alpha_x)$ as well as the linear coupling $\beta$ and the energy splitting $\delta$, as shown in Fig. 2.

5. Lastly, there are two technical points, that provide constraints on the claim of "univeral topology", which need to be addressed. The authors use the function $F(w)$ to indicate a general nonlinearity and create a Lyapunov potential based on that, where Taylor expansion is then carried out. In this notation, a) when $F'(1) = 0$ but $F''(1) \neq 0$, then expansion of $V(r, \phi)$ does not have a quadratic term, but instead a cubic term with coefficient proportional to $F''(1)$. Due to this qualitative change, the $D_4^-$ unfolding and topology breaks down. The same applies for higher-order smooth functions $F(w)$ indicating higher-order nonlinearities. b) If the nonlinearities depend on the angle (not just $w$, but $\theta$ as well) or have non-diagonal

terms, then Lyapunov function existence is not given. (As an example, consider $F(w,\theta) = kw\cos\ \theta$, where one can see that $\partial_\theta(w^{-1}f_1)|_{EP} = 2\alpha k \neq \partial_w(-f_2)|_{EP} = 0$.)

In item 5(a), the reviewer correctly analyzes the case in which the first derivative vanishes, $F'(1) = 0$, and demonstrates that he/she is able to understand our analysis at a very deep level. Indeed, as we said in the original manuscript in the sentence immediately after Eq. (15) of the Supplementary Information, the leading-order term is $r^2, r = 1 - w$, if $F(w = 1) = 0$ and "its first derivative there is non-zero." And in the main text we said "... any diagonal linear or nonlinear perturbation that depends only on $w$ through a function $F(w)$ with $F(w) = 0$ and $dF(w)/dw \neq 0$ at the linear EP is in this category." The original manuscript thus implied that higher-order nonlinearities are included in our analysis only if they lead to a function $F(w)$ for which $F'(1) \neq 0$ (as discussed above, in the revised manuscript we were able to substantially broaden our claim and relax this restrictions made in the original manuscript). For us, it is encouraging to see that the reviewer has looked at our calculations in such great detail, because it not only shows that the reviewer is an expert in the field, but we take it to imply that the reviewer is genuinely interested in the subject, and that therefore we can expect more of the general readership of Nature Communications to be interested in our study.

And yes, in 5(b), the reviewer again provides a correct analysis and deep insight for the case that $F = F(w,\theta)$, which agrees with our statement in the original manuscript at the end of Sec. 1.B.1 of the Supplementary Information, where in the original Supplementary Information we said "With other forms of nonlinearities, e.g. $F$ complex, $F$ is a function of both $w$ and $\theta$, ... the eigenvector equations are in general not derivable from a Lyapunov potential."

But again, in the revised manuscript, we are able to clarify that basically all functions $F(w)$ that occur in physics (functions that are sufficiently smooth) lead to elliptic umbilic nonlinear EU structures, and we want to stress that our theory may still be valid, even if, for example, $F$ is a function of both $w$ and $\theta$. It is true that for those cases we have not been able to find the Lyapunov potential, but that doesn't mean that the Lyapunov potential does not exist. In fact, we believe that it is reasonable to assume that all nonlinearities added to our linear matrix will lead to elliptic umbilic EP structures. In the revised version of the manuscript, we say in Sec. 2 (using, for simplicity, the sufficient, but not necessary condition of *F* being analytic):

Since, in the linear case, we find the germ to be that of an elliptic umbilic catastrophe, we conjecture that all nonlinearities that are real or complex functions of the variables *w* and $\theta$ defining the eigenvectors, and that are analytic in a neighborhood around the linear EPs, will lead to locally elliptic umbilic singularity sets.

In order to further illustrate the extension beyond the Kerr-like nonlinearity, in the revised manuscript we now have the following paragraph in Sec. 1.2 (which was partly reproduced above, in item (3)):

Let us first note that the matrix in Eq. (1) is equivalent, as far as the topology of the EP object in parameter space is concerned, to one that contains self coupling ( $\alpha_c$ ) and cross coupling ($\alpha_x$) terms with quadratic nonlinearities:

$$H_1 = \begin{pmatrix} \delta + i\gamma + \alpha_c|x|^2 + \alpha_x|y|^2 & \beta \\ \beta & -\delta - i\gamma + \alpha_c|y|^2 + \alpha_x|x|^2 \end{pmatrix}.$$

with $\alpha = (\alpha_c - \alpha_x)/2$. The two matrices differ by a multiple of the unit matrix, and thus have the same eigenvectors while the eigenvalues differ only by a constant shift

$\bar{\alpha} = (\alpha_c + \alpha_x)/2$. Here, we assume the couplings $\alpha_c$ and $\alpha_x$ to be real-valued, but below we will see that they also can be imaginary, which in some applications can be related to gain saturation. We can generalize the matrix in Eq. (6) to allow for arbitrary smooth real valued nonlinearity functions $f(u)$

$$H_1 = \begin{pmatrix} \delta + i\gamma + \alpha_c f(|x|) + \alpha_x f(|y|) & \beta \\ \beta & -\delta - i\gamma - \alpha_x f(|x|) + \alpha_c f(|y|) \end{pmatrix}.$$

Similar to the case discussed above, we can substract a diagonal matrix that is a unit matrix multiplied by the (nonlinear) constant $g(\mathrm{x}) = \bar{\alpha}(f(|x|) + f(|y|))$, i.e. we define $H_1 = g(\mathrm{x})\hat{1} + H$, where $\hat{1}$ is the unit matrix, and obtain

$$H = \begin{pmatrix} \delta + i\gamma + \alpha(f(|x|) - f(|y|) & \beta \\ \beta & -\delta - i\gamma - \alpha(f(|x|) - f(|y|)) \end{pmatrix}.$$

As in the original manuscript, we write the nonlinearity in the form

$$\alpha(f(|x|) - f(|y|) = \alpha F(w)$$

with

$$F(w) = f\left(\frac{1}{\sqrt{1+w^2}}\right) - f\left(\frac{w}{\sqrt{1+w^2}}\right)$$

and now provide important examples at the end of Sec. 1.2:

Similar to the example of the quadratic nonlinearity discussed in the previous section, the EP is at $w = 1$. As mentioned at the beginning of Sec. 1, the condition for the elliptic umbilic singularity is that at the linear EP (where $w = 1$ in the case of symmetric matrices), the function $F(w)$ must be approximated by a power expansion. Therefore, important examples of functions with elliptic umbilic singularity sets include all power functions $f(u) = u^n$ with nonzero exponent, $n \neq 0$, such as the quartic nonlinearity $f(u) = u^4$, and periodic functions such as $f(u) = \cos(u^2)$.

Furthermore, in Sec. 1.3 we consider four useful categories of the function, grouping them in terms of their low-order expansion behavior, and in Sec. 1.4 we state **that asymmetric matrices as well as matrices with complex nonlinear coefficients α are all covered by our theory** (i.e. they have elliptic umbilic EP structures). Algebraic details for those cases are given in the Supplementary Information.

We further clarify the terminology, and more importantly main claim of our manuscript, in the following new paragraph:

Interestingly, we see that the non-trivial topology of the set of EPs in the nonlinear parameters space (the parameter space that includes the nonlinearity $\alpha$, as shown in Fig. 2), is already fully determined by the EPs in the linear space (the subspace of parameters where the nonlinearity is zero). In other words, there are infinitely many possibilities for the EP topology in the nonlinear case (cf. Fig. 1b), but already from the properties of the linear EP alone we find that the topology must be the cone-like structure. At small nonlinearities, this structure is of the form of an elliptic umbilic, i.e. a cone with a deltoid cross section (and the cone-like structure can persist at larger nonlinearities, as in our example, preserving the topology of the elliptic umbilic but not its precise geometry). This is a remarkable statement, since usually one would try to find the set of EPs in the nonlinear parameters space by

searching or scanning the part of the parameter space where the nonlinearity is non-zero. To be more specific, if in Eq. (6) we set $\alpha = 0, \beta' = 0$ and $\delta = 0$, we are left with the so-called 'germ' (sometimes called degenerate core) of the catastrophe. For catastrophes with codimension up to 5 (called elementary catastrophes), the germs can be found in textbooks, e.g. [33, 75] and are reproduced in Supplementary Table I. Each germ gives an unambiguous assignment of the corresponding elementary catastrophe. Since, in our case, the Lyapunov potential (as a polynomial in the state variables given by the eigenvectors) that generates the eigenvector equations of the nonlinear eigenvalue problem exists, we can predict the topology of the EP singularity set in the full parameter space solely from the Lyapunov potential where all parameters (including the nonlinearity) have been set to zero. The precise geometric shape can change under smooth transformations of parameters, but its topological properties are determined by the universal unfolding (which is defined as the versal unfolding of minimal dimension, see, e.g., p. 621 of Ref. [75]) of the elliptic umbilic singularity.

Due to these concerns, we feel that the claim of "universal topology" is not justified in the case presented in the manuscript, much less for larger-dimensional systems.

We want to stress first that, in the revised manuscript, the nonlinearity function $F(w)$ can basically be any function (as long as it is sufficiently smooth at the linear EP), which means that basically **all functions *F(w)* that are of practical importance in physics are included in our analysis.** But regarding the specific use of the word "universal" we would like to note the following Nat. Comm. policy.

Delineating the meaning of a technical term used in the title (here 'universal') through explanations in the abstract and the main text is in line with common practice in Nat. Comm. and other high-profile journals. For example, the recent paper by Kilic et al., ***Universal*** *symmetry-protected persistent spin textures in noncentrosymmetric crystals*, Nat. Comm. (2025) 16:7999 (2025), clarifies in the abstract that the word 'universal' in the title is to be understood **with the exception** of all crystals in the space group P1, even though they are noncentrosymmetric. Another example is ***Universality*** *of Dicke superradiance in arrays of quantum emitters* by Masson and Asenjo-Garcia, Nat. Comm. 13:2285 (2022), where the analysis is **restricted** to Markovian system, leaving out all systems with quantum memory effects.

Finally, we provide two tables meant to illustrate the generality (universality) of our results which, in the revised manuscript, is discussed in much greater detail and - we believe - made much clearer:

| Categories now explicitly mentioned | Original manuscript | Revised manuscript |
|---|---|---|
| $F(1) = 0$ and $F'(1) = 0$ | ✗ | ✓ |
| $F(1) = 0$ and $F'(1) \neq 0$ | ✓ | ✓ |
| $F(1) \neq 0$ and $F'(1) = 0$ | ✗ | ✓ |
| $F(1) \neq 0$ and $F'(1) \neq 0$ | ✗ | ✓ |
| Here, the nonlinearity in H is assumed to be written as $\alpha F(w)$ and $F'(w) = dF/dw$ | | |

| Examples within category $F$(1)=0 and $F'$(1)≠0 | Original manuscript | Revised manuscript |
|---|---|---|
| quadratic nonlinearity $f(u) = u^2$ | ✓ | ✓ |
| power law nonlinearity $f(u) = u^n$ $(n \neq 0)$<br>oscillatory nonlinearity $f(u) = cos(u^2)$ | ✗ | ✓ |
| nonlinear coefficient $\alpha$ real | ✓ | ✓ |
| nonlinear coefficient $\alpha$ complex | ✗ | ✓ |
| symmetric matrix, off-diagonal $b = c$ real | ✓ | ✓ |
| symmetric matrix, off-diagonal $b = c$ complex | ✗ | ✓ |
| asymmetric matrix, off-diagonal $b, c$ arbitrary complex | ✗ | ✓ |
| Here, the nonlinearity in H is assumed to be written as $\alpha F(w) = \alpha(f(\lvert x \rvert) - f(\lvert y \rvert))$<br>The off-diagonal elements are written as $\begin{pmatrix} \cdots & b \\ c & \cdots \end{pmatrix}$ | | |

We hope that we have sufficiently addressed all the Reviewer's concerns and he/she will now recommend publication of our manuscript.

Reviewer #5 (Remarks to the Author):

**We thank the reviewer for reviewing our manuscript.**

I co-reviewed this manuscript with one of the reviewers who provided the listed reports. This is part of the Nature Communications initiative to facilitate training in peer review and to provide appropriate recognition for Early Career Researchers who co-review manuscripts.

**<u>Response to reviewers' reports, manuscript NCOMMS-25-60031-T, Kwong et al., *Universal Topology of Exceptional Points in Nonlinear Non-Hermitian Systems*</u>**

We would like thank Reviewers #3 and #4  for reviewing our manuscript again.

Below, we respond to all comments, questions and concerns in the reviews. The original reports are reproduced in blue font, our response is typed in black font.

Accompanying this response is a version of our manuscript where all substantive changes are highlighted in red. The Supplementary Information has not changed.

**Reviewer #3 (Remarks to the Author):**

The paper has been significantly revised and substantially improved. The comments raised in the reports have been addressed. I would have preferred a more detailed discussion of the limitations of the potentiality condition, rather than the current brief statement (“Since, in our case, the Lyapunov potential … exists…”). However, since the authors explicitly acknowledge these limitations while still claiming “universality,” it is probably acceptable to leave the text as it is.

I would also have liked to see more physical background, beyond simply referring to papers [86–89], as the current approach may limit the audience. Nevertheless, I am prepared to leave this decision to the authors.

I would give the authors the opportunity to implement these comparatively minor changes, although the paper could also be recommended for publication in its current form.

We thank Reviewers #3 for recommending the manuscript to be published with optional minor changes. The changes that the Reviewer proposes are indeed constructive and helpful, and we are happy to implement them in order to improve the quality of our paper.

In the introduction, we now say (changes highlighted in red):

"...In this paper, we demonstrate that important insight can be obtained on the distribution of EPs in parameter space, especially for nonlinear eigenvalue problems, with implications for photonic systems with Kerr-type nonlinearity, atomic [71, 72] and polariton condensates [73], superfluids [74] and coupled ring or micropillar optical [75] and electric resonators [66]. Additional novel applications, such as robust wireless power transfer and enhanced frequency-comb generation are discussed in Ref. [76]...."

and we repeat those references in various places. Refs. [75,76,99] were added to further broaden the scope and consider the most recent progress in the field. We believe that it now becomes clearer that our mathematical conclusions apply to basically all current implementations of non-Hermitian systems with nonlinearity and exceptional points. Cases where the Lyapunov potential does not exist are discussed in the Methods section and the Supplemenet.  The word "universality" in the title refers to a "large class" (quote from the abstract) that is mathematically defined in the paper and that, as far as we know, covers all relevant physical systems that have

been discussed in the literature. It also includes all systems that all reviewers have specifically mentioned or asked about during the reviews process.

In response to both, Reviewer #3 and #4, we have clarified the conjecture formulated in the Conclusion section.

"...Since, in the linear case, we find the germ to be that of an elliptic umbilic catastrophe, we conjecture that all nonlinearities that are real or complex functions of the variable w (and possibly both w and θ) defining the eigenvectors, and that are analytic in a neighborhood around the linear EPs and fulfill the relaxed vanishing-curl condition (see discussion following Eq. (16) in Methods), will lead to locally elliptic umbilic singularity sets. ..."

and, also in the Conclusion, added

"...symmetric matrices with complex parameters in the off-diagonal elements also belong to this class. To the best of our knowledge, the class of matrices covered by our theory includes most, if not all, nonlinear EP systems that are presently discussed in the literature (Kerr-type nonlinearity, atomic and polariton condensates, superfluids, and coupled ring or micropillar optical and electric resonators). Further extensions of the class of matrices with the elliptic umbilic topological structure may be possible...."

**Reviewer #4 (Remarks to the Author):**

EDITOR'S EMAILS: Please see the attached PDF file.
AUTHOR'R REMARK: For the purposes of this response, we have transcribed the pdf file containing the review to MS Word format.

The revised version improves the universality claim by explicitly deriving the result in the Lyapunov construction and clarifying the analytic assumptions near the linear EP. But the reduction to catastrophe theory requires existence of a scalar Lyapunov potential whose critical points coincide with the eigenvector solutions. This demands an integrability condition on the reduced vector field as $\partial_\theta f = \partial_w g$.

Analyticity alone does not imply this property. Here is a simple counter-example: Under an analytic perturbation $\tilde{f} = f + \varepsilon p, \tilde{g} = g + \varepsilon q$ with analytic $p, q$, choosing for example $p(w, \theta) = w\theta$ and $q = 0$ yields $\partial_\theta \tilde{f} - \partial_w \tilde{g} = \varepsilon w \neq 0$, violating exactness despite analyticity.

Therefore the universality statement that all nonlinearities analytic in ( $w, \theta$ ) near the EP lead to elliptic-umbilic singularity sets is not justified or applicable.. Analyticity alone does not imply either of these properties. The universality claim must therefore be conditioned by (i) an exactness condition ensuring existence of a Lyapunov potential and (ii) nondegeneracy conditions on the third-order Taylor expansion of the Lyapunov potential around the linear EP with the restriction to the integrable analytic subclass explicitly constructed in the manuscript. However, doing so reduces the weakens the impact of the "universal" claim that seeks to connect exceptional points to catastrophe theory.

The main issue raised by the Reviewer is 'exactness', for which the reviewer gives a condition ("... $\partial_\theta f = \partial_w g$ ... ") and a detailed counterexample ("...example $p(w,\theta) = w\theta$ and $q = 0$ yields $\partial_\theta \tilde{f} - \partial_w \tilde{g} = \varepsilon w \neq 0$, violating exactness despite analyticity....").

This issue is discussed in detail in the Methods section of our manuscript. The term 'exact' is commonly used in the context of differential forms, where it refers to a form that can be written as the exterior derivative of another form. In our paper, we don't use that formalism. The precise mathematical condition in the context of our paper is given in Eq. (16) of Methods, and we refer to it as the 'vanishing curl' condition. As stated in that section, the 'vanishing curl' condition does not strictly apply in our case; only a relaxed version of that condition applies to us.

Since the Methods section has not changed since the original submission of the manuscript, the Reviewer's observation of the 'exactness' condition does not imply new restrictions of our analysis.

The Reviewer's statement that

"... Therefore the universality statement that *all* nonlinearities analytic in ( $w, \theta$ ) near the EP lead to elliptic-umbilic singularity sets is not justified or applicable "

is correct, but this statement is not made in our manuscript. We assume that the Reviewer's statement was motivated by the conjecture made at the end of the Conclusion section. We stress that a conjecture is not the same as a claim. A conjecture needs to have a reasonable basis, which it does in our case. The claims that we make are based on a specific mathematical analysis of nonlinearity functions $F(w)$. It is possible that for nonlinearities of the form $F(w,\theta)$ additional restrictions regarding the third-order Taylor expansion apply (item (ii)). Our manuscript does not include those functions. In order to further clarify this, we added the following:

We also note that *F* might be a function of both *w* and $\theta$, and the eigenvector equations may not be derivable from a Lyapunov potential. General bifurcation theory may be needed to study the conditions under which we still have an elliptic umbilic singularity.

This does not change the large class of matrices that we have identified in our manuscript to lead to elliptic umbilic singularities.

Even though item (ii) of Reviewer #4 appears to be limited to the case of $F(w,\theta)$, we note that for the case of nonlinearity functions $F(w)$, details of the third-order Taylor expansion are given on p. 4 of the Supplement. Again, the Reviewer's comments do not imply new restrictions of our analysis.

The word "universality" in the title refers to a "large class" (quote from abstract) that is mathematically defined in the paper and that, as far as we know, covers all relevant physical systems that have been discussed in the literature. It also includes all systems that all reviewers have specifically mentioned or asked about during the reviews process.

In response to both, Reviewer #3 and #4, we have clarified the conjecture formulated in the Conclusion section:

"...Since, in the linear case, we find the germ to be that of an elliptic umbilic catastrophe, we conjecture that all nonlinearities that are real or complex functions of the variable w (and possibly both w and θ) defining the eigenvectors, and that are analytic in a neighborhood around the linear EPs and fulfill the relaxed vanishing-curl condition (see discussion following Eq. (16) in Methods), will lead to locally elliptic umbilic singularity sets. ..."

and, also in the Conclusion, added

"...symmetric matrices with complex parameters in the off-diagonal elements also belong to this class. To the best of our knowledge, the class of matrices covered by our theory includes most, if not all, nonlinear EP systems that are presently discussed in the literature (Kerr-type nonlinearity, atomic and polariton condensates, superfluids, and coupled ring or micropillar optical and electric resonators). Further extensions of the class of matrices with the elliptic umbilic topological structure may be possible...."

We also made a minor correction to the axis label in Fig. 1c,d.

Report on "Universal topology of exceptional points in nonlinear non-Hermitian systems" by N.H. Kwong et al.

In this article, the authors consider a two-state dynamical system described by a non-Hermitian generator of time evolution H. The eigenvalue problem for such nonlinear H is recast in terms of Lyapunov function, whose vanishing gradient generates the eigenvalue equations and whose vanishing Hessian gives the exceptional point degeneracies of the nonlinear Hamiltonian. By using Kerr-type (cubic) nonlinearity, the Lyapunov function is constructed and shown to match the elliptic-umbilic category from catastrophe theory. The authors obtain analytical and numerical results for the evolution of nonlinear EPs as a function of the nonlinearity strength. Then the authors claim that the results are generalizable to other nonlinearities.

The paper is well written, with extensive supplementary material and references. The key, interesting aspect here is mapping the eigenvalue equation for the 2-dimensional complex eigenvector, characterized by $(w, \theta)$, into Lyapunov function with no reference to the eigenvalue $E$, so that the results in the classical Catastrophe theory with two variables (and number of parameters, three in this case, corresponding to dimensionless coupling $\beta/\gamma$, dimensionless detuning $\delta/\gamma$, and dimensionless nonlinearity $\alpha/\gamma$). This mapping onto specific, elliptic umbilic topology is clear for the Kerr-nonlinearity problem they have considered; however, we have several concerns regarding the claim of "universal topology". The following need to be addressed before the paper can be further considered:

1. The discussion is focused on eigenvalue degeneracy criterion. When claiming EPs, as understood in the non-Hermitian community, the athors should also show corresponding eigenvector coalescence. In linear models, it is easy to create examples where a 3-fold eigenvalue degeneracy has two independent eigenvectors, thereby having only EP2.

2. The authors create a Lyapunov potential that generates the eigenvalue equation. However, the uniqueness of this potential is not addressed. Specifically, different potentials can give rise to different Hessians and thereby potentially different EP landscapes. The authors should address this in the main text. In dissipative systems, many different Lagrangians can give rise to the same equations of motion (for example, mass+spring system with time-dependent mass vs. Bateman oscillators, both give rise to the equations of motion for a damped harmonic oscillator.)

3. The paper discussion is rigorous for cubic nonlinearities in the Hermitian part of the Hamiltonian that arise from the Kerr effect. However, they do not encompass major physically relevant or mathematically interesting cases. Notable absences include the following. a) The gain-saturation nonlinearity that shows up in the anti-Hermitian part of the Hamiltonian, and is asymmetric meaning only arises for the gain side, not the loss side. b) In the Hermitian part, higher-order nonlinearities – particularly a quartic one – are relevant; see, for example, nonlinear optics by Robert Boyd chapter 1. c) In a system like a nonlinear pendulum, or its quantum equivalent in a superconducting circuit, the nonlinearity is a periodic function of the dynamical variable.

4. In the current manuscript, the results provided are symmetric in sign of nonlinearity, $\alpha/\gamma$. However, in the Kerr model for the continuum nonlinear Schrodinger equation, positive and negative signs refer to physically different effects (focusing vs. non-focusing). The authors should comment on the seeming presence of this symmetry.

5. Lastly, there are two technical points, that provide constraints on the claim of "univeral topology", which need to be addressed. The authors use the function $F(w)$ to indicate a general nonlinearity and create a Lyapunov potential based on that, where Taylor expansion is then carried out. In this notation, a) when $F'(1) = 0$ but $F''(1) \neq 0$, then expansion of $V(r, \phi$ does not have a quadratic term, but instead a cubic term with coefficient proportional to $F''(1)$. Due to this qualitative change, the $D_4^-$ unfolding and topology breaks down. The same applies for higher-order smooth functions $F(w)$ indicating higher-order nonlinearities. b) If the nonlinearities depend on the angle (not just $w$, but $\theta$ as well) or have non-diagonal terms, then Lyapunov function existence is not given. (As an example, consider $F(w, \theta) = kw \cos\theta$, where one can see that $\partial_\theta(w^{-1} f_1)|_{EP} = 2\alpha k \neq \partial_w(-f_2)|_{EP} = 0$.)

Due to these concerns, we feel that the claim of "universal topology" is not justified in the case presented in the manuscript, much less for larger-dimensional systems.

The revised version improves the universality claim by explicitly deriving the result in the Lyapunov construction and clarifying the analytic assumptions near the linear EP. But the reduction to catastrophe theory requires existence of a scalar Lyapunov potential whose critical points coincide with the eigenvector solutions. This demands an integrability condition on the reduced vector field as $\partial_\theta f = \partial_w g$.

Analyticity alone does not imply this property. Here is a simple counter-example: Under an analytic perturbation $\tilde{f} = f + \varepsilon p$, $\tilde{g} = g + \varepsilon q$ with analytic $p, q$, choosing for example $p(w, \theta) = w\theta$ and $q = 0$ yields $\partial_\theta \tilde{f} - \partial_w \tilde{g} = \varepsilon w \neq 0$, violating exactness despite analyticity.

Therefore the universality statement that *all* nonlinearities analytic in $(w, \theta)$ near the EP lead to elliptic-umbilic singularity sets is not justified or applicable.. Analyticity alone does not imply either of these properties. The universality claim must therefore be conditioned by (i) an exactness condition ensuring existence of a Lyapunov potential and (ii) nondegeneracy conditions on the third-order Taylor expansion of the Lyapunov potential around the linear EP with the restriction to the integrable analytic subclass explicitly constructed in the manuscript. However, doing so reduces the weakens the impact of the ”universal” claim that seeks to connect exceptional points to catastrophe theory.
